# Correlative Imaging Platform Linking Taste Cell Function to Molecular Identity

**DOI:** 10.1002/advs.202511309

**Published:** 2025-11-30

**Authors:** Sungho Lee, Minjae Kim, Gha Yeon Park, Jubeen Yoon, Kunyoo Shin, Chang Ho Sohn, Myunghwan Choi

**Affiliations:** ^1^ School of Biological Sciences Seoul National University Seoul 08826 Republic of Korea; ^2^ The Institute of Molecular Biology and Genetics Seoul National University Seoul 08826 Republic of Korea; ^3^ Center for Nanomedicine Institute for Basic Science Seoul 03722 Republic of Korea; ^4^ Graduate Program of Nano Biomedical Engineering (NanoBME) Advanced Science Institute Yonsei University Seoul 03722 Republic of Korea; ^5^ Graduate School of Medical Science and Engineering Korea Advanced Institute of Science and Technology Daejeon 34051 Republic of Korea

**Keywords:** correlative imaging, in vivo imaging, single‐cell analysis, spatial transcriptome, taste

## Abstract

Understanding the physiology of taste cells requires multifaceted cellular information, ranging from gene expression to functional responses. Various experimental approaches are available to obtain each biological information, such as in situ hybridization for gene transcription and in vivo microscopy for functional responses. However, correlative acquisition of genetic and functional information at the single‐cell level has not yet been achieved for taste cells, limiting a comprehensive understanding of functional and genetic cell subtypes. Here, we developed a correlative imaging platform to link functional responses of taste cells to their molecular identity. This platform acquires functional imaging data and in situ hybridization or immunofluorescence data from the same region‐of‐interest in intact taste buds, using near‐infrared branding (NIRB) to ensure spatial correspondence between live and fixed tissues. As a proof‐of‐principle, we demonstrated that sour cells in vivo specifically express the molecular marker, carbonic anhydrase IV (CA4), at both transcriptional and translational levels. Harnessing this platform, we revealed that sweet/umami dual‐tuned responses in gustatory nerves are mainly mediated by synaptic input from taste cells co‐expressing all taste receptor type 1 (Tas1R) subtypes, suggesting a combinatoric encoding of preferred taste qualities. We anticipate that our correlative platform facilitates a deeper understanding of taste information processing.

## Graphical abstract

A correlative imaging platform is developed to study how individual taste cells respond to different taste qualities. By linking cellular activity with molecular identity and environmental context, dual‐tuned taste cells capable of detecting both sweet and umami stimuli are identified. This platform provides mechanistic insight into how discrete taste qualities can be encoded and processed by the same population of taste cells.

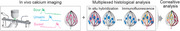

## 1 Introduction

The taste system detects and processes diverse stimuli to guide feeding behavior. This process begins with taste cells, which are housed in taste buds distributed across the tongue and palate.^[^ ^1^ ^]^ Upon stimulation, taste cells activate intracellular signaling pathways and transmit signals to connected gustatory afferents.^[^ ^2^ ^]^ These cells have been classified into several subtypes based on morphological, genetic, and functional features.^[^ ^3^ ^]^ This subtype classification has relied on multifaceted approaches, including functional recordings and postmortem molecular analyses.^[^ ^4^, ^5^ ^]^ While informative, these methods typically examine isolated aspects of taste transduction, limiting our understanding of how anatomical, genetic, and molecular mechanisms together shape peripheral taste sensation.

Recent advances in single‐cell RNA sequencing (scRNA‐seq) have enabled transcriptomic profiling of hundreds to thousands of individual cells, revealing a new dimension of biological complexity.^[^ ^6^, ^7^ ^]^ To directly link gene expression with cellular function, scRNA‐seq has been combined with activity‐dependent labeling techniques such as Fos‐TRAP,^[^ ^8^ ^]^ PhotoSeq,^[^ ^9^ ^]^ and photoconversion.^[^ ^10^ ^]^ To further preserve spatial context, there have been efforts to utilize single‐molecule fluorescence in situ hybridization (smFISH) and multiplexed immunofluorescence (IF) with live functional imaging. These imaging‐based approaches enable high‐resolution molecular analysis while maintaining anatomical localization, thereby linking function to transcriptomic or proteomic features in situ. Increasingly, multimodal approaches integrating cellular activity, mRNA expression, and protein localization within their native spatial context are being employed.^[^ ^11^, ^12^, ^13^ ^]^ When unified into a consolidated platform, these methods can overcome the limitations of individual techniques and provide a more comprehensive understanding of cell function.^[^ ^14^, ^15^, ^16^ ^]^

In parallel with advances in molecular technologies, efforts have continued to capture the functional dynamics of intact taste cells in their native milieu. Notably, in vivo functional imaging became feasible with the development of a microfluidic‐based tongue imaging window (µTongue).^[^ ^17^, ^18^ ^]^ This platform revealed complex functional heterogeneity even among genetically classified cell subtypes, underscoring the necessity of directly linking physiological activity with molecular identity. For instance, within sweet‐responsive type II taste cells, some display heightened sensitivity to artificial sweeteners, while others preferentially respond to natural sugars.^[^ ^19^ ^]^ Moreover, response duration and adaptation rates vary substantially among cells of the same subtype.^[^ ^20^ ^]^

To date, however, correlative integration of functional‐molecular co‐localization at the single‐cell level has not yet been achieved on taste cells due to the absence of a structural fiducial marker for linking in vivo to ex vivo and the unique composition of the tongue tissue.^[^ ^21^ ^]^ The tongue epithelium is composed of a highly hydrophobic, keratinized epithelium with dense extracellular matrix.^[^ ^22^ ^]^ This unique composition impedes the use of available tissue processing tools developed for the central nervous system. In addition, taste cells on the tongue epithelium suffer from structural deformations during tissue processing, further complicating the integration of molecular and functional data (Table S1, Supporting Information). Consequently, this limitation hinders a comprehensive understanding of functional and genetic cell subtypes involved in peripheral taste processing.

To address these challenges, we developed a correlative imaging platform that links the functional activity of taste cells with their molecular identity. This platform combines microfluidics‐based in vivo tongue imaging system (µTongue),^[^ ^17^, ^18^, ^20^, ^23^ ^]^ with subsequent smFISH or IF on the same region‐of‐interest in intact taste buds. As validation, we demonstrated that sour‐responsive taste cells in vivo selectively express the canonical marker, CA4, at both mRNA and protein levels. Leveraging this platform, we further identified that sweet/umami dual‐tuned afferent responses are primarily driven by synaptic input from taste cells co‐expressing all Tas1R receptor subtypes, suggesting that the peripheral taste system, particularly with respect to appetitive taste qualities (sweet and umami), cannot be fully explained by the widely accepted labeled‐line model, which posits that each taste cell encodes only a single taste quality.

## 2 Results

### 2.1 Development of Correlative Imaging Workflow

To investigate taste‐evoked responses of taste cells, we utilized the Pirt‐GCaMP6‐tdTomato mouse model (Figure S1, Supporting Information), which labels chemosensory taste cells, including type II (sweet, umami, bitter) and type III (sour) taste cells,^[^ ^5^ ^]^ as well as afferent nerves innervating the taste buds.^[^ ^20^ ^]^ This model offers a distinct advantage by allowing simultaneous monitoring of calcium dynamics in both chemosensory taste cells and afferent nerves in response to taste stimuli through volumetric time‐series imaging of the taste bud.

**Figure 1:**
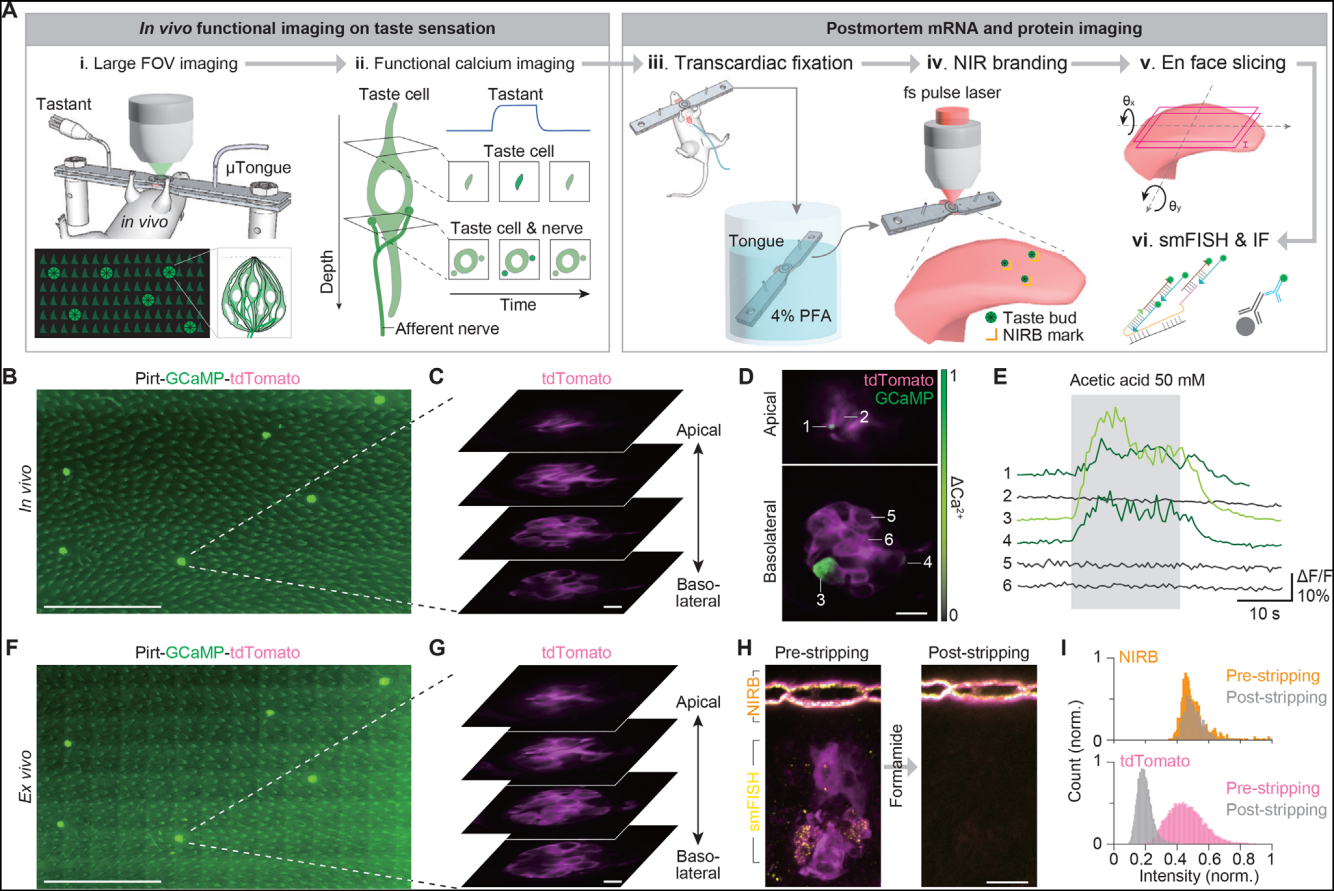
Correlative imaging pipeline. A) Schematic of the correlative workflow. (i) A mouse is mounted on a µTongue device, and the relative positions of taste buds are acquired through a large field‐of‐view image. (ii) The 3D structure of the taste bud is acquired, followed by functional screening using the µTongue device. (iii) While attached to the device, the mouse undergoes cardiac perfusion, and the excised tongue tissue is fixed in PFA for over 48 h. (iv) After fixation, NIRB markers are generated around the identical taste buds, which are screened during in vivo imaging, before removing the device. (v) After removing the device, the tongue is sectioned into slices <170 µm thick in the en face (horizontal) direction. (vi) Then, smFISH and/or IF are performed on the specimen. B) Representative large field‐of‐view image from an in vivo imaging session. Scalebar: 500 µm. C) Representative 3D structure images of a taste bud highlighted with a dashed line in (B). Scalebar: 5 µm. D) The identical taste bud in (C) during in vivo functional imaging upon sour stimuli, showing calcium responses from the apical (top) side and the basolateral (bottom) side of the taste cell. Scalebar: 10 µm. E) Time‐lapse GCaMP6 intensity traces corresponding to the numbered cells indicated in (D). F) large field‐of‐view image in (A) after sectioning. Scalebar: 500 µm. G) 3D structure image of identical taste buds shown in (C). Scalebar: 10 µm. H) Representative images of pre‐ and post‐stripping of probes. Note that, while the fluorescence signal from both smFISH and fluorescence reporter was diminished, the auto‐fluorescence induced by NIRB persisted. I) Quantitative comparison of signal changes in NIRB and fluorescence reporter after treatment with 90% formamide.

For establishing a correlative platform for multimodal profiling, we developed an integrated experimental workflow that combines functional imaging and genetic screening. We employed the µTongue system to track individual taste cells throughout both in vivo and ex vivo imaging steps. The workflow was carefully optimized to preserve spatial integrity, enabling seamless transition from in vivo functional imaging to ex vivo molecular analyses and ensuring accurate single‐cell‐level correlative observation (**Figure** **1**A; Figure S2, Supporting Information). We first acquired a large field‐of‐view image prior to functional taste screening, generating a reference map for accurate localization of individual taste buds based on their relative positions (Figure 1B). We then selected at least five taste buds and extracted their relative coordinates from the large field‐of‐view image. Subsequently, a high‐resolution 3D structural image was acquired to enable single‐cell level investigation (Figure 1C). Since intracellular calcium responses in type II taste cells are primarily confined to the apical side, whereas afferent nerves exhibit calcium activity in the basolateral side, we acquired pseudo‐volumetric images of the taste bud at three to four axial positions. This approach allows us to capture calcium dynamics in both apical and basolateral regions, thereby clearly distinguishing the activity of taste cells from that of afferent nerves (Figure 1D,E).

Taste cells located on the surface of the tongue are particularly susceptible to structural deformation during histological sample preparation. To mitigate this, we performed cardiac perfusion with the µTongue device mounted on the tongue. Post‐fixation of the excised tongue was also performed with the µTongue device in place to ensure minimal structural distortion. After fixation, we disassembled the tongue from the device and identified the same taste buds based on their relative position (Figure 1F) and their three‐dimensional structural images (Figure 1G).

Multi‐round smFISH requires probe stripping and re‐probing.^[^ ^24^ ^]^ Although DNase I has been successfully used to remove smFISH probes in the brain,^[^ ^16^, ^25^ ^]^ the highly hydrophobic keratin layer on the surface of the tongue significantly impairs stripping efficiency, as evidenced by the incomplete removal of Hoechst‐labeled nuclear signals at concentrations effective in brain tissue (Figure S3, Supporting Information). To address this issue, we optimized a cost‐effective probe stripping strategy using 90% formamide. Although this method effectively removes probes, it also potentially denatures fluorescent proteins expressed in taste cells, leading to quenching of both tdTomato and GCaMP6.^[^ ^26^, ^27^ ^]^ Unfortunately, this fluorescence quenching also leads to loss of relative position over multiple rounds of hybridization. To address this issue, we performed NIRB^[^ ^28^ ^]^ to generate robust autofluorescence landmarks for tracking the same taste buds of interest across imaging sessions (Figure 1H). Unlike endogenous fluorescent proteins, NIRB‐induced autofluorescence is resistant to formamide‐induced quenching, ensuring reliable taste bud identification (Figure 1I). After NIRB marking, we trimmed excess tongue tissue to improve histological processing efficiency and ensure uniform chemical penetration (Figure S2, Supporting Information).

Considering that taste cells are tightly entangled within taste buds,^[^ ^1^, ^29^ ^]^ the confined microenvironment of taste buds necessitates precisely regulated ATP‐mediated signaling, where both the intrinsic molecular identity of individual cells and their spatial context critically modulate signal specificity and dynamics. To elucidate this fine‐tuned functional orchestration, full spatial sampling is required to resolve interactions within the densely packed cellular architecture of taste buds.^[^ ^29^, ^30^ ^]^ In response to this challenge, we sectioned the NIRB‐marked tongue coaxially (en face) at a thickness of 170 µm, approximately twice the thickness of taste cells. While spatial transcriptomic methods such as RNAscope and Multiplexed Error‐Robust Fluorescence In Situ Hybridization (MERFISH) achieve simultaneous detection of dozens to thousands of genes at a single‐cell resolution, the available sample thickness is restricted to 10–20 µm.^[^ ^16^, ^25^ ^]^ To overcome this constraint, we employed smFISH with hairpin chain reaction (HCR), which exhibits superior hybridization efficiency in thick tissue.^[^ ^16^ ^]^

### 2.2 Data Processing

**Figure 2:**
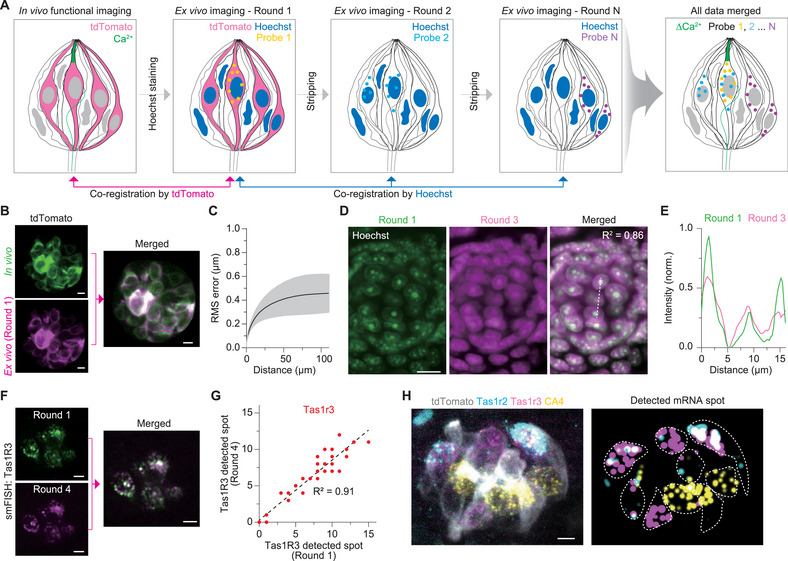
Data processing workflow. A) Schematic of image registration workflow. B) Representative images showing registration between the in vivo image and the first round histological image via fluorescent reporter (tdTomato). The same taste cells were spatially aligned using B‐spline registration. Scalebar: 5 µm. C) RMS error measurement of in vivo versus the first round fluorescent reporter in identical taste cells. The black line indicates the mean, and the gray shaded area denotes the standard error mean (SEM). (*n* = 3 mice, 16 taste buds). D) Representative multi‐round rigid‐body registration based on Hoechst staining. Mean image correlation coefficient was 0.86. Scalebar: 10 µm. E) Line profile of Hoechst signals from the first and third rounds, indicated by the yellow dashed line in (D). Peak signals showed a 2‐3 pixel difference (<1 µm). F) Representative images of the same mRNA FISH performed in the first and fourth. Scalebar: 5 µm. G) Spot count comparison from identical taste cells that express Tas1R3 at the first round and fourth round. H) Multi‐round image registration and mRNA spot visualization. Scalebar: 5 µm. Each color indicates a targeted mRNA (Cyan: Tas1R2, magenta: Tas1R3, and yellow: CA4).

Functional and multi‐round histological imaging generate large datasets, often several gigabytes per experiment. To efficiently integrate spatial information across multiple imaging rounds, we have developed a data processing pipeline that constructs single‐cell multimodal datasets. The reference channel for multi‐round image registration was lost due to endogenous fluorescent protein quenching during probe stripping (Figure 1H). To address this, we performed nuclear staining using Hoechst in all imaging rounds and used the first round histological images as a universal reference for registration. Specifically, the initial fluorescence signal (tdTomato) served as an in vivo reference, while the nuclear signal was used for image registration in subsequent rounds (**Figure** **2**A). Consistent with previous studies, we observed minor structural deformation after paraformaldehyde (PFA) fixation.^[^ ^31^ ^]^ To correct this, we performed B‐spline‐based non‐rigid registration to align the in vivo functional data with the first round in situ structural images of the taste buds (Figure 2B; Figure S4, Supporting Information). To quantify the degree of distortion, we computed the deformation vector field and measured the root‐mean‐square (RMS) error. The resulting error was minimal (<0.5% of the measured distance), rendering accurate tracking of individual taste cells (Figure 2C). Because negligible structural deformation was observed in the nuclear‐stained images from multiple rounds of results, we then employed rigid registration to align images acquired over multiple rounds. To validate the performance of rigid registration, we calculated the Pearson correlation coefficient between the first round and subsequent rounds (Figure 2D) and measured fluorescence intensity line profiles (Figure 2E).

A major limitation of multi‐round smFISH is the progressive decrease in calling rates for mRNA spot detection as the number of hybridization cycles increases.^[^ ^25^ ^]^ In our experimental design, at least three rounds of imaging were required to image all marker genes associated with the five basic taste qualities while minimizing spectral overlap. Specifically, the first round was limited to one marker gene, while up to three marker genes could be detected in each subsequent round. To assess mRNA stability across rounds, we re‐probe Tas1R3 in both rounds 1 and 4. Analysis of the detected spots confirmed that mRNA signals remained stable and detectable through the fourth round (Figure 2F,G). After round‐to‐round image registration, we conducted cell segmentation to quantitatively assess mRNA expression levels at single‐cell resolution. For this, we employed VAST Lite 1.4.0^[^ ^32^ ^]^ for cell segmentation and Airlocalize^[^ ^33^ ^]^ for semi‐automated smFISH spot detection. Individual detected mRNA spots were quantified using the digital HCR (dHCR) approach, in which spot counting was performed based on the number of discrete fluorescent dots. In the first round, tdTomato fluorescence uniformly represents the cytosol of individual taste cells, enabling precise cell segmentation. After registering images using Hoechst staining, we confirmed that cytosolic boundaries remain well‐defined, even when merged with Hoechst signals. Therefore, we first generated the segmentation mask based on the first round image, and this mask was applied to all subsequent rounds (Figure S3, Supporting Information). By integrating both software tools, we successfully achieved molecular identification at single‐cell resolution (Figure 2H).

### 2.3 Demonstration of Correlative Imaging on Sour‐Responsive Cells

**Figure 3:**
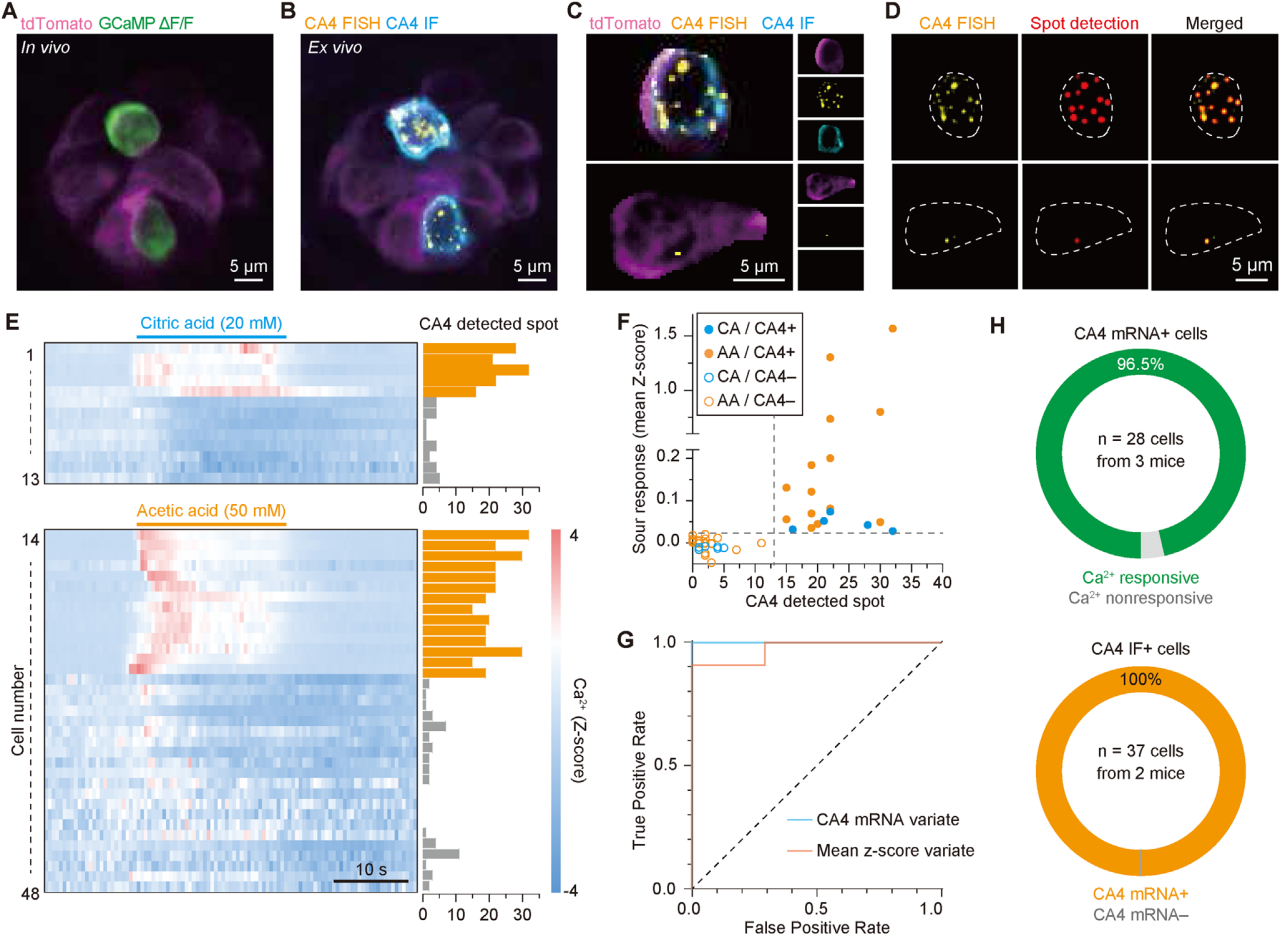
Correlative imaging demonstration on sour‐responsive cell. A) Representative calcium activity in Pirt‐labeled taste cells within a taste bud during 50 mm acetic acid stimulation. Scalebar: 5 µm B) Post hoc multiplex imaging of the same taste bud (round 1: CA4 mRNA smFISH, round 2: CA4 IF) in (A). Scalebar: 5 µm. C,D) Single‐cell analysis and spot quantification from (B). Scalebar: 5 µm. E) Correlative integration in sour‐responsive cells. Both sour tastants reliably elicit calcium responses, which were exclusive to cells highly expressing CA4 mRNA. F) CA4 IF+ cells show a positive correlation to cells with high detection of CA4 mRNA spots and responsivity to sourness. Each circle represents an individual taste cell (blue: citric acid, yellow: acetic acid; filled: IF‐positive, empty: IF‐negative). G) ROC for CA4 mRNA spots (blue) and mean Z‐score (red) as univariate classifiers. H) Pie chart showing sour‐responsive cells distribution stratified by CA4 mRNA count (top) and CA4 IF+ cell proportion (bottom).

To validate the correlative platform and data processing pipeline, we examined type III taste cells, which respond to sour stimuli and express CA4. Type III cells were chosen because of their relatively sparse distribution (≈20% of the taste bud) and their characteristic global calcium influx into the cell body, which helps to distinguish them from other taste cell types.^[^ ^34^ ^]^ As shown in Figure 1D, we applied sour taste stimuli (20 mm citric acid or 50 mm acetic acid) to Pirt‐labeled taste cells and identified sour‐responsive cells via calcium imaging, followed by smFISH for CA4 mRNA and IF for CA4 protein, demonstrating that both mRNA and protein expression are restricted to sour‐responsive cells (**Figure** **3**A,B). For quantitative analysis at single‐cell resolution, we generated a single‐cell mask based on the tdTomato fluorescence from the first round (Figure 3C). By conducting integrated single‐cell analysis, we quantified CA4 mRNA expression in individual CA4 protein+ and CA4 protein− cells (Figure 3D). Quantitatively, correlative analysis using CA4 IF as a reference revealed a strong association among functional activity, protein, and mRNA expression levels at single‐cell resolution. (Figure 3E). Integration of these quantitative data sets, we confirmed that the correlative platform effectively links calcium activity with molecular markers at single‐cell resolution. To further evaluate these associations, we performed receiver operating characteristic (ROC) analysis to quantify the relationships among functional activity, protein, and mRNA expression levels. Notably, CA4 IF+ and IF− cells were clearly distinguished by mean Z‐score responses to sour stimulation, with a classification threshold of 0.23. Similarly, CA4 IF+ and IF− cells are classified using a threshold of 13 CA4 smFISH spots, validating the strong correlation between molecular markers and functional activity (Figure 3F,G). Consistent with previous studies,^[^ ^35^ ^]^ these findings demonstrate that our correlative imaging platform successfully integrates functional and molecular information. By consolidating multimodal measurements into a single experimental framework, our approach provides a comprehensive understanding of taste cell function at the molecular level (Figure 3H).

### 2.4 Correlative Mapping of Spatial and Molecular Factors Driving Dual‐Tuned Taste Signaling

**Figure 4:**
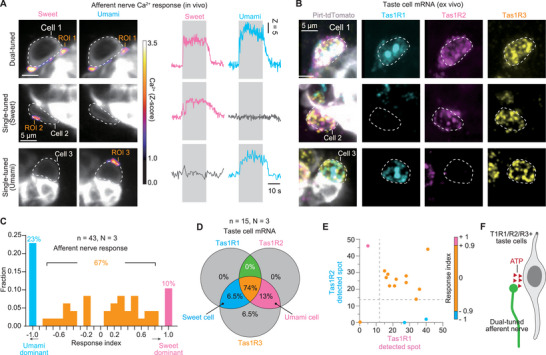
Dual‐tuned sweet–umami afferent activity originates from intrinsic receptor expression profiles of single taste cells. A) Representative calcium activity in the basolateral afferent nerves to sweet and/or umami tastes (left) and afferent nerve calcium trace (right). White dashed circles indicate individual taste cells. ROIs denote the magnitude of calcium responses in their synaptic afferent nerves. Scalebar: 5 µm. B) The composite fluorescent images of 2‐multiplexing round showing expression of mRNA of the identical taste cells as presented in (A), using a correlative platform (round 1 for Tas1R3, and round 2 for Tas1R1 and Tas1R2). Each color represents a distinctly targeted smFISH probe. Scalebar: 5 µm. C) Histogram showing the distribution of mean calcium activity index under tastant stimuli. +1 indicates sweet‐cells (magenta), −1 indicates umami‐cells (cyan), and responses near 0 (yellow) are classified as dual‐tuned responses. D) Pie chart showing the number of detected spots for each gene (Tas1R1, Tas1R2, Tas1R3) in cells adjacent to the afferent nerves analyzed in (C). E) Scatter plot of the correlation between the number of detected mRNA spots for Tas1R1 and Tas1R2 and the response index. F) Working model of dual‐tuned responses in afferent nerves, which are mediated by single taste cells that express multiple taste receptors.

Transcriptomic profiling of Tas1Rs expressing taste cells^[^ ^36^ ^]^ indicated that a subset of these cells co‐express sweet (Tas1R2/R3) and umami (Tas1R1/Tas1R3) receptors. In addition, structural reconstruction data showed some afferent nerves form multiple synapses with taste cells,^[^ ^29^ ^]^ suggesting potential convergence of sweet and umami pathways. To further explore this phenomenon, we investigated dual‐tuned sweet‐umami responses within taste buds using our correlative platform. We applied sweet and umami taste stimuli in vivo using the µTongue: sweet (30 mm acesulfame potassium; AceK) and umami (50–150 mm monopotassium glutamate; MPG, and 1–3 mm inosine monophosphate; IMP). Consistent with previous findings, we identified sweet‐tuned, umami‐tuned, and dual‐tuned cells. Most responsive afferent nerves were found adjacent to Pirt‐expressing taste cells, likely due to wavy junctions^[^ ^29^ ^]^ (**Figure** **4**A). Heterodimerization of Tas1R genes is essential for the detection of both sweet and umami taste substances (sweet: Tas1R2/Tas1R3, umami: Tas1R1/Tas1R3).^[^ ^37^ ^]^ To clarify this phenomenon, we performed smFISH in two or three rounds. We then conducted histological analysis of taste cells located near afferent nerves responding to sweet and/or umami stimuli (Figure 4B). Among these cells, mRNA expression patterns are classified into three groups: umami cells (Tas1R1+/R3+), sweet cells (Tas1R2+/R3+), and cells expressing both markers (Tas1R1+/R2+/R3+). To correlate taste responses with gene expression patterns, we first classified afferent nerve responses using a response index formula.

(1) $$\mathrm{Resp}\mathrm{onse}\mathrm{index}=\frac{\mathrm{Mean}\mathrm{Zsco}\mathrm{re}\left[\mathrm{sweet}\right]-\mathrm{Mean}\mathrm{Zsco}\mathrm{re}\left[\mathrm{umami}\right]}{\mathrm{Mean}\mathrm{Zsco}\mathrm{re}\left[\mathrm{sweet}\right]+\mathrm{Mean}\mathrm{Zsco}\mathrm{re}\left[\mathrm{umami}\right]}$$

The mean Z‐score reflects the average calcium activity during each tastant stimulus. Based on the baseline calcium activity, we set the cutoff for distinguishing responsive and non‐responsive populations at a mean Z‐score of 1 (Figure S5, Supporting Information). If the mean Z‐score did not exceed this threshold, the value was set to 0 (non‐responsive). Cases where both Z‐scores are zero were excluded from the data set. Using the response index, we defined response values between −1 and 1 as indicative of multi‐tuned afferent nerves. Single‐tuned responses corresponded to values of −1 or 1, whereas intermediate values within this range were classified as multi‐tuned. Because the response amplitudes varied across different tastants and our analysis was focused on the cell responsiveness rather than response magnitude, we considered all intermediate values between −1 and 1 as multi‐tuned responses (Figure 4C). To quantitatively assess the relationship between afferent nerve responses and the receptor profiles of adjacent taste cells, we examined the mRNA spots detected by smFISH in taste cells adjacent to the 43 afferent nerves used for response index measurements. Since type II cells include populations expressing not only Tas1Rs but also Tas2Rs, determining their receptor expression based on IF results alone is more challenging than that of type III cells. Given that CA4+ cells are distinct from sweet/umami− responsive cells, we used the number of Tas1Rs mRNAs in CA4+ cells as a criterion to establish a negative control (Figure S6, Supporting Information). Consistent with afferent nerve responses, we identified 15 taste cells located near responsive afferent nerves that show an abundant presence of all three receptor types (Figure 4D). Furthermore, visualization of the response index along with sweet (Tas1R2) and umami (Tas1R1) receptor expressions reveals that taste responses are determined by the level of receptor mRNA presence (Figure 4E). In contrast, about 10% dual‐tuned afferent nerves were found in close proximity to both sweet cells and umami cells. In this case, the afferent nerves also respond to both sweet and umami stimuli (Figure S7, Supporting Information). This spatial arrangement suggests that multi‐tuning may not be solely attributed to the intrinsic properties of the individual taste cells. Instead, ATP, the neurotransmitter released upon sweet and umami activation, may diffuse into shared extracellular spaces. However, the majority of afferent nerves (≈90%) are associated with taste cells expressing all three receptor types (Tas1R1+/R2+/R3+), supporting the hypothesis that the dual‐tuned responses of these nerves are primarily driven by the intrinsic properties of the dual‐tuned receptor cells themselves (Figure 4F).

In conclusion, our results indicate that the majority (∼90%) of sweet‐umami dual‐tuned responses in afferent nerves are determined by the mRNA expression patterns (Tas1R1+/R2+/R3+) of the contacted taste cells, while a smaller subset (≈10%) arises from ATP spillover in cells of close‐proximity interaction. These results showed that the signal processing of these appetitive taste qualities (sweet and umami) follows not only the labeled‐line model but also combinatory processing. This highlights the complex interplay of molecular and spatial factors in shaping taste perception.

## 3 Discussion

Our correlative platform represents a significant advance in the field of taste research by integrating the functional activity and transcriptional profiles of individual taste cells at the single‐cell level. Importantly, this novel approach has enabled a comprehensive investigation of the functional and molecular dynamics of peripheral taste cells, overcoming the limitations imposed by the complex physical and structural nature of the tongue.

Our investigation of the dual‐tuned responses of afferent nerves has provided valuable insights into sweet and umami taste activity. Notably, a significant number of afferent nerves showed calcium responses to both sweet and umami stimuli, suggesting that these signals are often mutually responsive rather than independently processed. This integration appears to be primarily influenced by the intrinsic receptor expression patterns of the associated taste cells, as demonstrated by our correlative analysis. Many taste cells located near dual‐tuned afferent nerves were found to co‐express receptor combinations, particularly Tas1R1+/R2+/R3+, enabling them to respond to both taste qualities. This receptor expression profile presumably underpins the observed convergence of sweet and umami signals in afferent nerves, suggesting that combinatorial encoding arises at the peripheral receptor level for these appetitive taste qualities. Despite the perceptual distinctiveness of sweet and umami tastes, the concurrent activation of afferent nerves by both stimuli suggests that taste sensation involves a combinatorial coding mechanism, where the multi‐tuned activity of afferent nerves shapes the overall perceptual outcome. This finding implies that taste perception is not strictly limited to discrete pathways but involves overlapping and integrative processing of taste information. Moreover, the observation of dual‐tuned responses driven by either receptor co‐expression or ATP spillover highlights the critical role of molecular and spatial factors in shaping afferent nerve activity. Although our data strongly suggest that sweet‐umami dual‐tuned responses derive from dual‐tuned taste receptor cells, we cannot exclude the possibility that these dual‐tuned responses derived from taste buds may be modulated during transmission to higher‐order centers, such as the nucleus tractus solitarius (NTS) and gustatory cortex.^[^ ^38^, ^39^, ^40^, ^41^ ^]^ This underscores the importance of considering both biological and technical limitations when interpreting neuronal activity patterns. This mechanism likely extends beyond sweet and umami to include combinations of other taste qualities, further emphasizing the complexity and integration involved in peripheral taste information processing. Future studies that incorporate enhanced sensitivity in signal detection and refined methodologies for tracking neuronal propagation dynamics in the geniculate ganglion will also be critical to fully elucidate the mechanisms underlying dual‐tuned taste responses.

Although this study did not directly investigate such combinations, there is potential for further improvement by integrating our approach with other existing methods. First, we used GCaMP6 to measure the activity of taste cells. Although calcium is a versatile secondary messenger involved in a variety of intracellular signaling pathways,^[^ ^42^ ^]^ its low temporal resolution and inability to specifically detect activation driven by specific ions and calcium‐independent pathways.^[^ ^43^ ^]^ Moreover, interpreting cellular responses solely based on calcium signaling may overlook critical components of intercellular communication.^[^ ^20^ ^]^ This suggests that integrating additional sensing modalities, such as GRAB sensors^[^ ^44^, ^45^ ^]^ could provide a more comprehensive understanding of intra‐ or inter‐cellular signaling mechanisms in the taste bud. In addition, combining advanced optical imaging modalities^[^ ^23^, ^46^, ^47^, ^48^, ^49^, ^50^ ^]^ may enable the detection of concentration‐dependent cellular responses, which are currently obscured by refractive index changes that mediate via focal shifts.

In our correlative studies, we have faced several limitations and challenges. We observed a progressive decrease in Hoechst signal, used as a registration guide, which remains an ongoing challenge in multiplexing research.^[^ ^25^ ^]^ While extensive optimization has been performed in the central nervous system, similar advances have not been made yet for the peripheral tissues, including the tongue. The implementation of spectral‐unmixing^[^ ^51^, ^52^ ^]^ methods may allow the simultaneous detection of multiple mRNAs in fewer cycles, while further optimization of probe stripping protocols will be essential to advance future studies. Finally, the dense distribution of taste cells in a confined space highlights the need for improved optical resolution. Enhancing spatial resolution and addressing issues such as smFISH probe aggregation are critical to obtain more accurate quantitative mRNA data. Super‐resolution imaging or expansion‐based methods offer potential solutions. In this study, we attempted to incorporate tissue expansion into our correlative platform, building on previous research.^[^ ^53^, ^54^, ^55^ ^]^ However, the conventional method of using proteinase K (ProK) at 37 °C failed to effectively digest the tongue tissue. When treated with ProK and incubated at 60 °C for one day, the tissue exhibited partial swelling and became transparent. Microscopic observation using a digestion buffer without ProK revealed that approximately 60% of the tissue showed uniform expansion (Figure S8, Supporting Information). In contrast, expansion trials in deionized water resulted in non‐uniform swelling, with the structures on the surface remaining intact. This incomplete digestion of the hydrophobic surface likely contributed to significant structural deformation of the tissue. Due to the low mRNA integrity observed at 60 °C (half‐life: 2.3–0.3 days depending on length),^[^ ^56^ ^]^ we did not further optimize this method at this stage. Nevertheless, incorporating tissue expansion techniques in future studies could improve our understanding of the taste system. In parallel with advances in image registration and the establishment of robust fiducial markers for in vivo taste cell structures, these approaches will facilitate the application of thin‐section histology within our platform. This, in turn, will allow integration with high‐throughput biomolecular platforms such as MERFISH and label‐free mass spectrometry imaging.^[^ ^57^, ^58^ ^]^ Furthermore, the adaptable framework of our correlative platform holds promise for application to other fields, such as the immune system and taste disorders. The highly motile and functionally diverse nature of the immune system presents unique challenges for integrated molecular‐functional profiling, particularly in capturing dynamic cell movements.^[^ ^59^ ^]^ In addition, the functional‐molecular mechanisms underlying taste disorders remain unknown. Dysgeusia, a representative taste disorder reported in patients undergoing chemotherapy, has been attributed to the inhibition of taste cell proliferation.^[^ ^60^ ^]^ However, the lack of appropriate experimental tools has limited efforts to directly link chemotherapy‐induced dysfunction with molecular alterations in mRNA or protein expression, or to explain how a reduction in taste cells affects taste quality. We believe that our approach offers new opportunities to address these gaps by directly linking functional responses of taste cells to key molecular changes, as well as spatial alterations in taste cells and their synaptic partner afferent nerves.

## 4 Conclusion

This study presents a correlative imaging platform that enables an integrated understanding of taste cell function and its molecular identity at single‐cell resolution. By combining in vivo calcium imaging, smFISH, and IF, we directly coupled functional responses of taste cells to their gene and protein expression profiles. Our approach revealed a strong correlation between CA4 expression and cellular responsiveness to sour taste stimulation, and further provided direct evidence of sweet–umami dual‐tuned nerve responses mediated by individual taste cells co‐expressing Tas1R family genes. These findings show that the peripheral taste system may not strictly adhere to the labeled‐line theory but rather involves a more integrated cellular mechanism in processing taste information. The platform developed here offers a powerful tool for dissecting the molecular and functional architecture of the taste system and holds potential for adaptation to other tissues for multimodal cellular analysis. Overall, our correlative platform presents a novel multi‐modal imaging data acquisition tool, and the incorporation of additional sensing modalities with advanced imaging techniques will further enhance our understanding of taste coding and its broader relevance in sensory biology.

## 5 Experimental Section

### Animal

All mice were housed with littermates in groups of two to five in a reverse day/night cycle and provided ad libitum access to food and water. Pirt‐GCaMP6f‐tdTomato mice aged 8 to 16 weeks were obtained by crossing PIRT‐IRES‐Cre (a line well established for targeting peripheral taste bud cells and gustatory geniculate ganglion neurons, provided by Xinzhong Dong at Johns Hopkins University) and CAG‐floxed‐GCaMP6f‐tdTomato (#031968, Jackson Laboratory). Both sexes of mice were used, and there was no significant difference between males and females. All animal experiments were performed in compliance with institutional guidelines and were approved by the Institutional Animal Care and Use Committee of Seoul National University (SNU‐240612‐2).

### Solutions

Artificial saliva and tastants prepared in artificial saliva were used for functional imaging. Composition of artificial saliva is as follows: 2 mm NaCl, 5 mm KCl, 3 mm NaHCO_3_, 3 mm KHCO_3_, 0.25 mm CaCl_2_, 0.25 mm MgCl_2_, 0.12 mm K_2_HPO_4_, 0.12 mm KH_2_PO_4_, and 1.8 mm HCl (pH: 7.4–7.6). Tastants were prepared by dissolving a panel of solutes in artificial saliva at the following concentrations: sweet, 30 mm AceK; sour, 10–20 mm citric acid or 50 mm acetic acid; umami, 50–150 mm MPG and 1–3 mm, and IMP. The detailed information is provided in Table S2 (Supporting Information).

### In Vivo Imaging

The mouse was mounted on the µTongue imaging chamber (µTongue Microfluidics, ALA scientific) following the procedures described previously.^[^ ^17^, ^18^, ^20^, ^23^ ^]^ Briefly, anesthesia was induced by intraperitoneal injection of a mixture of ketamine (100 mg∙kg^−1^) and dexmedetomidine (1 mg∙kg^−1^). After confirming deep anesthesia via toe pinch reflex, the scalp and periosteum of the mouse were carefully removed by using surgical scissors and a cotton swab. A custom‐designed head‐fixing apparatus was attached to the exposed cranium using medical adhesive (Loctite 4161, Henkel Corporation) and dental resin (#1223146, DenKist) to minimize head movement. The µTongue chamber itself was assembled by gluing the top and bottom pieces with a thermoplastic resin to form a microfluidic channel, which was connected to an eight‐channel fluidic delivery system (Octaflow II, ALA Scientific) via an eight‐port manifold (MPP‐8, Harvard Apparatus). The outlet of the microfluidic channel was linked to a syringe pump to maintain a constant flow rate of approximately 300 µL∙min^−1^. The tongue was gently extended using a cotton swab and plastic tweezers. The ventral surface of the tongue and the lower lip were affixed to the bottom side of the µTongue using medical adhesive. The exposed dorsal surface of the tongue was then kept moist with artificial saliva to preserve tissue viability.

After preparation, the mouse was mounted onto the stage of either a spinning‐disk confocal microscope (CSU‐W1, Yokogawa) or a multiphoton microscope (Bergamo II, Thorlabs). A 10× 0.45 NA objective (MRD00105, Nikon) was used to acquire large field‐of‐view images. The edges of the imaging window on the mouse tongue were selected as the boundary for tile‐scanning. Following tile‐scanning, individual images were stitched using the built‐in function of the NIS software (Nikon) or the Fiji plugin, BigStitcher. For functional imaging, the number of z‐planes was set to 3–5, covering the full depth at which taste buds are located. Subsequently, taste buds were imaged using a 40× 0.8 NA objective (MRD07420, Nikon) for in vivo calcium imaging. Functional imaging involved simultaneous acquisition of time‐lapse and z‐stack series. During the first 20 s, artificial saliva was applied to establish the GCaMP6 baseline signal. Taste solutions were then delivered for the following 20 s, followed by reapplication of artificial saliva for 50 s to wash out the previous stimulus before the next tastant was introduced.

### Fixation

After in vivo imaging, mice were transferred to a fume hood for cardiac perfusion, with the tongue remaining mounted on the µTongue. While under deep anesthesia, the mouse was transcardially perfused with 4% PFA in 0.1 m phosphate‐buffered saline (PBS). The posterior portion of the tongue was then carefully dissected, and the entire tongue, along with the µTongue, was fixed in 4% PFA at 4 °C for 48 h.

### Near‐Infrared Branding

To re‐identify identical taste buds of interest after probe stripping, a photo‐oxidation‐based labeling method was utilized, NIRB for the chemically formed fluorescence that is far more stable than fluorescence protein. The fixed tongue, remaining affixed to the µTongue, was re‐mounted onto the multiphoton microscope (Bergamo II, Thorlabs) equipped with a 920 nm femtosecond fiber laser (FemtoFiber ultra 920, TOPTICA) to generate NIRB. To facilitate accurate identification of relative spatial positions, a 10 × 0.45 NA air objective (MRD00105, Nikon) was employed, which provides a large field of view. Because the fluorescence reporter remained detectable during PFA fixation, identical taste buds could be re‐accessed by comparing their relative positions with large‐field‐of‐view images acquired during in vivo imaging sessions. The scan path was then manually drawn adjacent to the target taste buds using the built‐in function of the image acquisition software (ThorimageLS, Thorlabs), with the branding position and depth defined at the nearest papillae while maintaining a minimum offset of 50 µm. The user‐defined ROI drawing was directly synchronized with the imaging system, ensuring that the scan path corresponded precisely to the image coordinates. After defining the scan path, the 920 nm laser was applied under the following conditions: average power of ≈200 mW at the back aperture, pixel dwell time of 50–500 µs, and iteration number of 2^6^–2^10^.^[^ ^28^ ^]^

### Correlative Sample Preparation

After completing NIRB, the tongue was detached from the µTongue. Subsequently, non‐imaged areas were excised using a razor blade under a surgical microscope (EZ4HD, Leica). The specimen was then sectioned en face using a vibratome (VT 1200s, Leica) at a thickness of ≈170 µm. The resulting sections were re‐examined under the microscope to ensure preservation of the structure by comparing them with the 3D images acquired during the in vivo imaging session. Following this verification, smFISH and/or IF procedures were conducted.

### Multi‐Round smFISH and Immunofluorescence

For smFISH and HCR, sections underwent pre‐incubation for permeabilization in 70% ethanol and were stored at room temperature for 1 h. Subsequently, the sections were incubated in 5X saline‐sodium citrate (SSC) buffer (AM9763, Thermo Scientific) and incubated at room temperature for 30 min. Following this, smFISH was performed according to the protocols provided by Molecular Instruments. In brief, the sections were incubated in probe hybridization buffer (BPH01625, Molecular Instruments) with the addition of 2 pmol of each probe set, and maintained at 37 °C for at least 16 h. Afterward, the sections were washed four times for 15 min each in wash buffer at 37 °C (BPW01825, Molecular Instruments), followed by four 15‐min washes in 5X SSC solution at room temperature. For the h1 and h2 hairpins, snap cooling was conducted in separate tubes at 95 °C for 90 s. Hybridization then proceeded in amplification buffer (BAM01725, Molecular Instruments) with 30 pmol of each hairpin added, and the samples were shielded from light for at least 14 h at room temperature. Finally, Hoechst (H3570, Thermo Scientific) was added to the 5x SSC buffer at a concentration of 10 µg∙mL^−1^ and stored at room temperature for one day before imaging. This method was consistently applied across all rounds. Detailed oligonucleotides and HCR amplifiers are listed in Table S3 (Supporting Information).

For the subsequent rounds of smFISH, probe stripping was performed by incubating sections in 90% formamide solution at 37 °C for 3 h, then washed in 5X SSC solution for 30 min at room temperature.

After completion of all smFISH rounds, sections were processed for free‐floating immunostaining. Initially, they were incubated in a blocking solution (008120, Thermo Scientific) for 2 h, followed by the addition of the primary antibody for a minimum duration of 2 days at room temperature, followed by four 15‐min washes in 1X PBS at room temperature. Secondary antibody incubation was performed at room temperature for 1 day followed by four 15‐min washes in 1X PBS at room temperature. Finally, Hoechst was added to the PBS at a concentration of 10 µg∙mL^−1^ and stored at room temperature for one day before imaging. Detailed IF products are listed in Table S4 (Supporting Information).

### Image Analysis Software

Images were visualized using either ImageJ (NIH) or NIS software (Nikon). The large field‐of‐view images were merged using BigStitcher in ImageJ or the built‐in function of the NIS software. B‐spline registration was performed using ITK‐elastix, while rigid body registration, spot detection, and cell masking were carried out in MATLAB (Mathworks). Detailed information about the software is listed in Table S5 (Supporting Information).

### Analysis of Functional Imaging Data

Calcium traces were analyzed using MATLAB built‐in functions and open‐source algorithms. Raw images were first motion‐corrected using a rigid motion correction algorithm (NoRMCorre) implemented in MATLAB.^[^ ^61^ ^]^ The calcium signals were then converted to ΔF/F and Z‐score by calculating the change in fluorescence ratio (green: GCaMP6f; red: tdTomato) relative to the baseline fluorescence ratio at selected ROIs, as previously described.^[^ ^62^, ^63^ ^]^ The responsive and non‐responsive nerve populations were determined based on whether the Z‐score during stimulation exceeded 2 SD of the baseline period. Image visualization and linear adjustment of brightness and contrast were performed using ImageJ (NIH) or Photoshop (Adobe).

### Image Registration

To register images of cellular taste responses acquired during in vivo imaging, the fluorescence signal was used from the tdTomato reporter obtained at the time of the initial smFISH as the reference image. This registration was performed using the ITK‐Elastix toolkit. To assess the extent of tissue deformation, non‐rigid (B‐spline) registration was applied, which generated a deformation vector field. This vector field was subsequently used to quantify registration errors across various length measurements.

To align images across multiple rounds of smFISH, nuclear images stained with Hoechst were used as landmarks. Nuclear stains from each round were registered to the first‐round image using built‐in MATLAB functions (imwarp), and the resulting transformation matrices were applied to the corresponding smFISH images. The accuracy of registration was evaluated by calculating image correlation coefficients and analyzing line profiles, also using MATLAB.

### Single Cell mRNA Spot Detection

To quantitatively measure mRNA at single‐cell resolution, cell segmentation was first performed using VAST Lite. The resulting segmented masks were then used to generate individual single‐cell images using custom MATLAB scripts. Spot detection across multi‐round images was carried out with Airlocalize,^[^ ^33^ ^]^ which includes a local background correction step that removes fluorescence adjacent to each detected spot. Briefly, a difference of Gaussians filter was applied to the images, followed by the subtraction of the global background. Local maxima exceeding a defined threshold within a 5‐pixel radius were then identified to estimate initial spot positions. In densely packed regions, mRNA spots were manually assigned to the cell structure that most closely matched their morphology.

### Statistical Analysis

Statistical analyses were performed using Prism 9 (GraphPad) and MATLAB (MathWorks). Parameters such as Z‐scores and area under the curve (AUC) were calculated in MATLAB. Heatmaps of processed trace data were generated using Prism 9. For comparison between two groups, either a paired test (Wilcoxon matched‐pairs signed rank test) or an unpaired test (Mann‐Whitney test) was applied, as appropriate. The Kolmogorov‐Smirnov test was used to compare cumulative distribution functions. All statistical tests were two‐sided, and p‐values less than 0.05 were considered statistically significant.

## Conflict of Interest

The authors declare no conflict of interest.

## Author Contributions

S.L., M.K., and G.Y.P. contributed equally to this work. S.L. and M.C. conceived and initiated this study. C.H.S. and M.C. supervised the research. S.L. developed a correlative platform. S.L. and M.K. optimized the image processing code. S.L., J.Y., and C.H.S. optimized a multiplex smFISH method on tongue tissue. K.S., M.K., and G.Y.P. conducted in vivo functional screening experiments. S.L., G.Y.P., C.H.S., and M.C. co‐wrote the paper with inputs from all authors.

## Supporting information



Supporting Information

## Data Availability

The data that support the findings of this study are available from the corresponding author upon reasonable request.

## References

[1] H. Lee , L. J. MacPherson , C. A. Parada , C. S. Zuker , N. J. P. Ryba , Nature 2017, 548, 330. 28792937 10.1038/nature23299 PMC5805144

[2] T. E. Finger , V. Danilova , J. Barrows , D. L. Bartel , A. J. Vigers , L. Stone , G. Hellekant , S. C. Kinnamon , Science 2005, 310, 1495. 16322458 10.1126/science.1118435

[3] P. A. S. Breslin , A. C. Spector , Curr. Biol. 2008, 18, R148. 18302913 10.1016/j.cub.2007.12.017

[4] S. C. Kinnamon , T. A. Cummings , S. D. Roper , Chem. Senses 1988, 13, 355.

[5] J. K. Roebber , S. D. Roper , N. Chaudhari , J. Neurosci. 2019, 39, 6224. 31171579 10.1523/JNEUROSCI.2367-18.2019 PMC6687907

[6] A. Zeisel , H. Hochgerner , P. Lönnerberg , A. Johnsson , F. Memic , J. van der Zwan , M. Häring , E. Braun , L. E. Borm , G. L. Manno , S. Codeluppi , A. Furlan , K. Lee , N. Skene , K. D. Harris , J. Hjerling‐Leffler , E. Arenas , P. Ernfors , U. Marklund , S. Linnarsson , Cell 2018, 174, 999. 30096314 10.1016/j.cell.2018.06.021 PMC6086934

[7] B. Tasic , Z. Yao , L. T. Graybuck , K. A. Smith , T. N. Nguyen , D. Bertagnolli , J. Goldy , E. Garren , M. N. Economo , S. Viswanathan , O. Penn , T. Bakken , V. Menon , J. Miller , O. Fong , K. E. Hirokawa , K. Lathia , C. Rimorin , M. Tieu , R. Larsen , T. Casper , E. Barkan , M. Kroll , S. Parry , N. V. Shapovalova , D. Hirschstein , J. Pendergraft , H. A. Sullivan , T. K. Kim , A. Szafer , et al., Nature 2018, 563, 72.30382198

[8] C. J. Guenthner , K. Miyamichi , H. H. Yang , H. C. Heller , L. Luo , Neuron 2013, 78, 773. 23764283 10.1016/j.neuron.2013.03.025 PMC3782391

[9] D. Lee , M. Kume , T. E. Holy , Science 2019, 366, 1384. 31831669 10.1126/science.aax8055 PMC7591936

[10] B. F. Fosque , Y. Sun , H. Dana , C. T. Yang , T. Ohyama , M. R. Tadross , R. Patel , M. Zlatic , D. S. Kim , M. B. Ahrens , V. Jayaraman , L. L. Looger , E. R. Schreiter , Science 2015, 347, 755. 25678659 10.1126/science.1260922

[11] S. Xu , H. Yang , V. Menon , A. L. Lemire , L. Wang , F. E. Henry , S. C. Turaga , S. M. Sternson , Science 2020, 370, abb2494. 10.1126/science.abb2494 PMC11938375 33060330

[12] L. J. von Buchholtz , N. Ghitani , R. M. Lam , J. A. Licholai , A. T. Chesler , N. J. P. Ryba , Neuron 2021, 109, 285. 33186546 10.1016/j.neuron.2020.10.028 PMC9909446

[13] N. Ghitani , L. J. von Buchholtz , D. I. MacDonald , M. Falgairolle , M. Q. Nguyen , J. A. Licholai , N. J. P. Ryba , A. T. Chesler , Nature 2025, 1. 10.1038/s41586-025-08875-6 PMC12222022 40269164

[14] M. Lipovsek , C. Bardy , S. J. Tripathy , C. R. Cadwell , K. Hadley , D. Kobak , J. Neurosci. 2021, 41, 937. 33431632 10.1523/JNEUROSCI.1653-20.2020 PMC7880286

[15] J. Fuzik , A. Zeisel , Z. Mate , D. Calvigioni , Y. Yanagawa , G. Szabo , S. Linnarsson , T. Harkany , Nat. Biotechnol. 2016, 34, 175. 26689544 10.1038/nbt.3443 PMC4745137

[16] Y. Wang , M. Eddison , G. Fleishman , M. Weigert , S. Xu , T. Wang , K. Rokicki , C. Goina , F. E. Henry , A. L. Lemire , U. Schmidt , H. Yang , K. Svoboda , E. W. Myers , S. Saalfeld , W. Korff , S. M. Sternson , P. W. Tillberg , Cell 2021, 184, 6361. 34875226 10.1016/j.cell.2021.11.024

[17] J. Han , M. Choi , bioRxiv 2018, 371682.

[18] J. Han , P. Choi , M. Choi , J. Vis. Exp. 2021, 2021. 10.3791/62361 33970147

[19] Z. Shi , W. Xu , L. Wu , X. Yue , S. Liu , W. Ding , J. Zhang , B. Meng , L. Zhao , X. Liu , J. Liu , Z.‐J. Liu , T. Hua , Nature 2025, 645, 801. 40555359 10.1038/s41586-025-09302-6

[20] G. Y. Park , G. Lee , J. Yoon , J. Han , P. Choi , M. Kim , S. Lee , C. Park , Z. Wu , Y. Li , M. Choi , Cell 2024, 188, 141. 39561773 10.1016/j.cell.2024.10.041

[21] R. B. Presland , B. A. Dale , Crit. Rev. Oral Biol. Med. 2000, 11, 383. 11132762 10.1177/10454411000110040101

[22] H. H. Bragulla , D. G. Homberger , J. Anat. 2009, 214, 516. 19422428 10.1111/j.1469-7580.2009.01066.x PMC2736122

[23] J. Han , S. Kim , P. Choi , S. Lee , Y. Jo , E. Kim , M. Choi , Biomed. Opt. Express 2021, 12, 5855. 34692220 10.1364/BOE.430643 PMC8515959

[24] E. Lubeck , A. F. Coskun , T. Zhiyentayev , M. Ahmad , L. Cai , Nat. Methods 2014, 11, 360. 24681720 10.1038/nmeth.2892 PMC4085791

[25] K. H. Chen , A. N. Boettiger , J. R. Moffitt , S. Wang , X. Zhuang , Science 2015, 348, 1360. 10.1126/science.aaa6090 PMC4662681 25858977

[26] G. Wei , L. Ma , M. Yan , X. Rong , Asian J. Chem. 2009, 21, 5299.

[27] J. Maillard , K. Klehs , C. Rumble , E. Vauthey , M. Heilemann , A. Fürstenberg , Chem. Sci. 2021, 12, 1352. 10.1039/d0sc05431c PMC8179231 34163898

[28] D. Bishop , I. Nikić , M. Brinkoetter , S. Knecht , S. Potz , M. Kerschensteiner , T. Misgeld , 2011, 8, 568. 10.1038/nmeth.1622 21642966

[29] C. E. Wilson , R. S. Lasher , R. Yang , Y. Dzowo , J. C. Kinnamon , T. E. Finger , J. Neurosci. 2022, 42, 804. 34876471 10.1523/JNEUROSCI.0838-21.2021 PMC8808728

[30] R. Ikuta , Y. Kakinohana , S. Hamada , Chem. Senses 2024, 49, bjae019. 38761122 10.1093/chemse/bjae019

[31] A. Idziak , V. V. G. K. Inavalli , S. Bancelin , M. Arizono , U. V. Nägerl , eNeuro 2023, 10, ENEURO.0104. 37709524 10.1523/ENEURO.0104-23.2023 PMC10521345

[32] D. R. Berger , H. S. Seung , J. W. Lichtman , Front. Neural Circuits 2018, 12, 88. 30386216 10.3389/fncir.2018.00088 PMC6198149

[33] T. Lionnet , K. Czaplinski , X. Darzacq , Y. Shav‐Tal , A. L. Wells , J. A. Chao , H. Y. Park , V. De Turris , M. Lopez‐Jones , R. H. Singer , Nat. Methods 2011, 8, 165. 21240280 10.1038/nmeth.1551 PMC3076588

[34] K. Lossow , I. Hermans‐Borgmeyer , M. Behrens , W. Meyerhof , Chem. Senses 2017, 42, 747. 29099943 10.1093/chemse/bjx048

[35] J. Chandrashekar , D. Yarmolinsky , L. Von Buchholtz , Y. Oka , W. Sly , N. J. P. Ryba , C. S. Zuker , Science. 2009, 326, 443. 19833970 10.1126/science.1174601 PMC3654389

[36] M. R. Kim , Y. Kusakabe , H. Miura , Y. Shindo , Y. Ninomiya , A. Hino , Biochem. Biophys. Res. Commun. 2003, 312, 500. 14637165 10.1016/j.bbrc.2003.10.137

[37] G. Q. Zhao , Y. Zhang , M. A. Hoon , J. Chandrashekar , I. Erlenbach , N. J. P. Ryba , C. S. Zuker , Cell 2003, 115, 255. 14636554 10.1016/s0092-8674(03)00844-4

[38] V. G. Sabater , M. Rigby , J. Burrone , J. Neurosci. 2021, 41, 5372. 34001627 10.1523/JNEUROSCI.2765-20.2021 PMC8221596

[39] I. H. Cho , L. C. Panzera , M. Chin , M. B. Hoppa , J. Neurosci. 2017, 37, 9519. 28871036 10.1523/JNEUROSCI.0891-17.2017 PMC6596771

[40] S. S. Goldstein , W. Rall , Biophys. J. 1974, 14, 731. 4420585 10.1016/S0006-3495(74)85947-3 PMC1334570

[41] N. Ofer , V. H. Cornejo , R. Yuste , iScience 2024, 27, 110884. 39346673 10.1016/j.isci.2024.110884 PMC11439538

[42] M. J. Berridge , M. D. Bootman , H. L. Roderick , Nat. Rev. Mol. Cell Biol. 2003, 4, 517. 12838335 10.1038/nrm1155

[43] K. Nomura , M. Nakanishi , F. Ishidate , K. Iwata , A. Taruno , Neuron 2020, 106, 816. 32229307 10.1016/j.neuron.2020.03.006

[44] Z. Wu , K. He , Y. Chen , H. Li , S. Pan , B. Li , T. Liu , F. Xi , F. Deng , H. Wang , J. Du , M. Jing , Y. Li , Neuron 2022, 110, 770. 34942116 10.1016/j.neuron.2021.11.027

[45] F. Sun , J. Zhou , B. Dai , T. Qian , J. Zeng , X. Li , Y. Zhuo , Y. Zhang , Y. Wang , C. Qian , K. Tan , J. Feng , H. Dong , D. Lin , G. Cui , Y. Li , Nat. Methods 2020, 17, 1156. 33087905 10.1038/s41592-020-00981-9 PMC7648260

[46] J. L. Fan , J. A. Rivera , W. Sun , J. Peterson , H. Haeberle , S. Rubin , N. Ji , Nat. Commun. 2020, 11, 1. 33243995 10.1038/s41467-020-19851-1 PMC7693336

[47] B. C. Chen , W. R. Legant , K. Wang , L. Shao , D. E. Milkie , M. W. Davidson , C. Janetopoulos , X. S. Wu , J. A. Hammer , Z. Liu , B. P. English , Y. Mimori‐Kiyosue , D. P. Romero , A. T. Ritter , J. Lippincott‐Schwartz , L. Fritz‐Laylin , R. D. Mullins , D. M. Mitchell , J. N. Bembenek , A. C. Reymann , R. Böhme , S. W. Grill , J. T. Wang , G. Seydoux , U. S. Tulu , D. P. Kiehart , E. Betzig , Science 2014, 346, 1297998. 10.1126/science.1257998 PMC4336192 25342811

[48] M. B. Bouchard , V. Voleti , C. S. Mendes , C. Lacefield , W. B. Grueber , R. S. Mann , R. M. Bruno , E. M. C. Hillman , Nat. Photonics 2015, 9, 113. 25663846 10.1038/nphoton.2014.323 PMC4317333

[49] V. Voleti , K. B. Patel , W. Li , C. Perez Campos , S. Bharadwaj , H. Yu , C. Ford , M. J. Casper , R. W. Yan , W. Liang , C. Wen , K. D. Kimura , K. L. Targoff , E. M. C. Hillman , Nat. Methods 2019, 16, 1054. 31562489 10.1038/s41592-019-0579-4 PMC6885017

[50] Z. Lu , S. Zuo , M. Shi , J. Fan , J. Xie , G. Xiao , L. Yu , J. Wu , Q. Dai , Nat. Biotechnol. 2025, 43, 569. 38802562 10.1038/s41587-024-02249-5 PMC11994454

[51] J. Seo , Y. Sim , J. Kim , H. Kim , I. Cho , H. Nam , Y. G. Yoon , J. B. Chang , Nat. Commun. 2022, 13, 2475. 35513404 10.1038/s41467-022-30168-z PMC9072354

[52] W. Hwang , T. Raymond , T. McPartland , S. Jeong , C. L. Evans , Commun. Biol. 2024, 7, 1012. 39154126 10.1038/s42003-024-06702-8 PMC11330493

[53] P. W. Tillberg , F. Chen , K. D. Piatkevich , Y. Zhao , C. J. Yu , B. P. English , L. Gao , A. Martorell , H. Suk , F. Yoshida , E. M. Degennaro , D. H. Roossien , G. Gong , U. Seneviratne , S. R. Tannenbaum , R. Desimone , D. Cai , E. S. Boyden , Nat. Biotechnol. 2016, 34, 987. 27376584 10.1038/nbt.3625 PMC5068827

[54] C. C. Yu , N. C. Barry , A. T. Wassie , A. Sinha , A. Bhattacharya , S. Asano , C. Zhang , F. Chen , O. Hobert , M. B. Goodman , G. Haspel , E. S. Boyden , Elife 2020, 9, 46249. 10.7554/eLife.46249 PMC7195193 32356725

[55] S. Alon , D. R. Goodwin , A. Sinha , A. T. Wassie , F. Chen , E. R. Daugharthy , Y. Bando , A. Kajita , A. G. Xue , K. Marrett , R. Prior , Y. Cui , A. C. Payne , C. C. Yao , H. J. Suk , R. Wang , C. C. Yu , P. Tillberg , P. Reginato , N. Pak , S. Liu , S. Punthambaker , E. P. R. Iyer , R. E. Kohman , J. A. Miller , E. S. Lein , A. Lako , N. Cullen , S. Rodig , K. Helvie , et al., Science 2021, 371, aax2656.

[56] U. Chheda , S. Pradeepan , E. Esposito , S. Strezsak , O. Fernandez‐Delgado , J. Kranz , J. Pharm. Sci. 2024, 113, 377. 38042343 10.1016/j.xphs.2023.11.023

[57] M. Angelo , S. C. Bendall , R. Finck , M. B. Hale , C. Hitzman , A. D. Borowsky , R. M. Levenson , J. B. Lowe , S. D. Liu , S. Zhao , Y. Natkunam , G. P. Nolan , Nat. Med. 2014, 20, 436. 24584119 10.1038/nm.3488 PMC4110905

[58] H. Zhang , K. H. Lu , M. Ebbini , P. Huang , H. Lu , L. Li , npj Imaging 2024, 2, 20. 39036554 10.1038/s44303-024-00025-3 PMC11254763

[59] J. L. Hor , R. N. Germain , Trends Cell Biol. 2022, 32, 406. 34920936 10.1016/j.tcb.2021.11.007 PMC9018524

[60] W. Ren , X. Cha , R. Xu , T. Wang , C. Liang , J. Chou , X. Zhang , F. Li , S. Wang , B. Cai , P. Jiang , H. Wang , H. Liu , Y. Yu , Theranostics 2023, 13, 2896. 37284449 10.7150/thno.81153 PMC10240818

[61] E. A. Pnevmatikakis , A. Giovannucci , J. Neurosci. Methods 2017, 291, 83. 28782629 10.1016/j.jneumeth.2017.07.031

[62] P. Zhou , S. L. Resendez , J. Rodriguez‐Romaguera , J. C. Jimenez , S. Q. Neufeld , A. Giovannucci , J. Friedrich , E. A. Pnevmatikakis , G. D. Stuber , R. Hen , M. A. Kheirbek , B. L. Sabatini , R. E. Kass , L. Paninski , Elife 2018, 7, 28728. 10.7554/eLife.28728 PMC5871355 29469809

[63] E. A. Pnevmatikakis , D. Soudry , Y. Gao , T. A. Machado , J. Merel , D. Pfau , T. Reardon , Y. Mu , C. Lacefield , W. Yang , M. Ahrens , R. Bruno , T. M. Jessell , D. S. Peterka , R. Yuste , L. Paninski , Neuron 2016, 89, 285. 26774160 10.1016/j.neuron.2015.11.037 PMC4881387

