# Supplementary material for: Correlative Imaging Platform Linking Taste Cell Function to Molecular Identity
Source: Adv Sci (Weinh). 2025 Nov 30;13(9):e11309. doi: 10.1002/advs.202511309 (PMC12904051; doi:10.1002/advs.202511309)
Supplement: Supplementary file 1 — Supporting Information [file ADVS-13-e11309-s001.docx]

Supporting Information

Correlative imaging platform linking taste cell function to molecular identity

Sungho Lee^1,2,†^, Minjae Kim^1,2,†^, Gha Yeon Park^1,2,†^, Jubeen Yoon^3,4,5^, Kunyoo Shin^1,2^, Chang Ho Sohn^5,^*****, Myunghwan Choi^1,2,^*

**This PDF file includes:**

Figs. S1 to S8

Tables S1 to S5


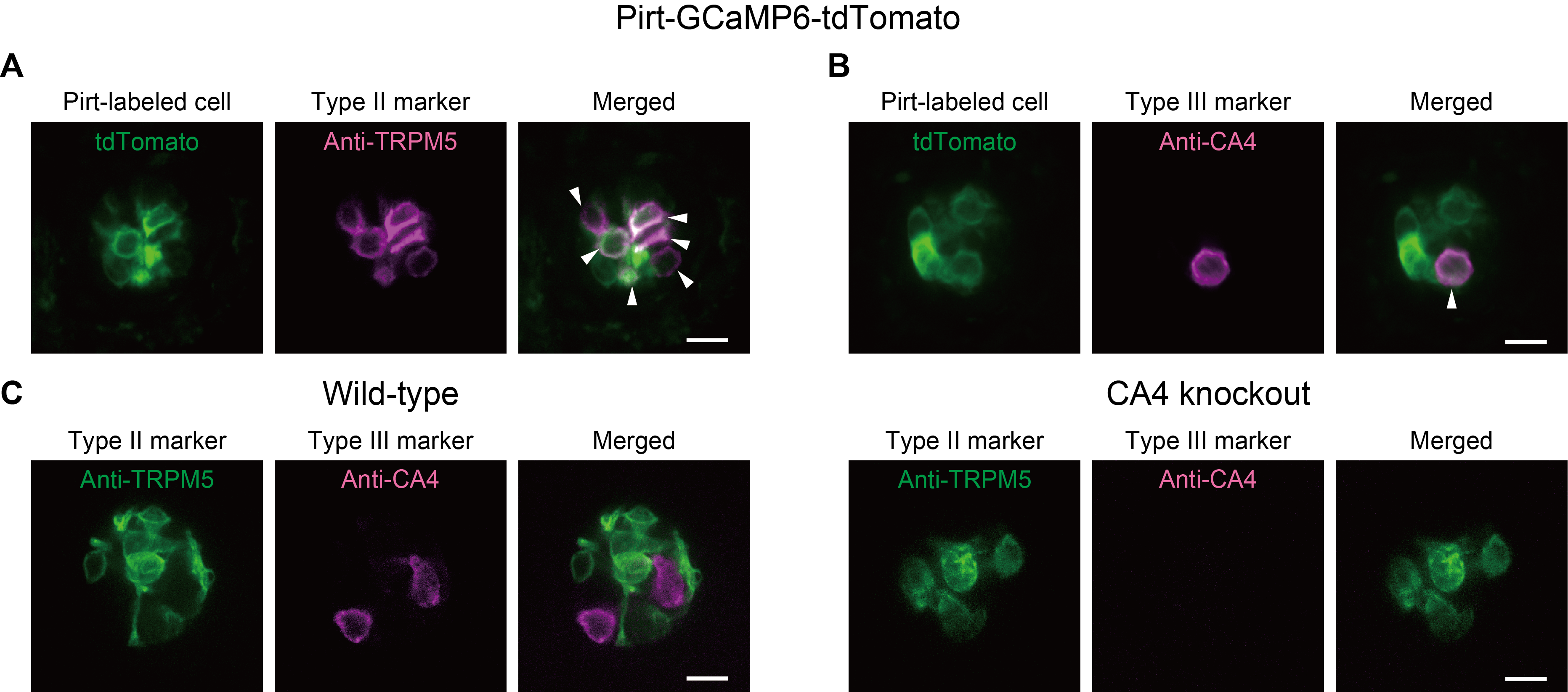


**Figure S1. Characterization of Pirt-GCaMP6s-tdTomato mouse and CA4 antibody validation**

**(A and B)** Representative fluorescence images of Pirt reporter mice showing TRPM5 (type II cell marker), and CA4 (type III cell marker). White arrowheads indicate cells co-expressing Pirt and the indicated immunofluorescence makers. **(C)** Representative images of a taste bud in wild-type (n = 1 mouse) and CA4 knockout mouse (n = 1 mouse), with TRPM5 (green) and CA4 (magenta) immunofluorescence staining. Scale bar in A-C: 20 µm.


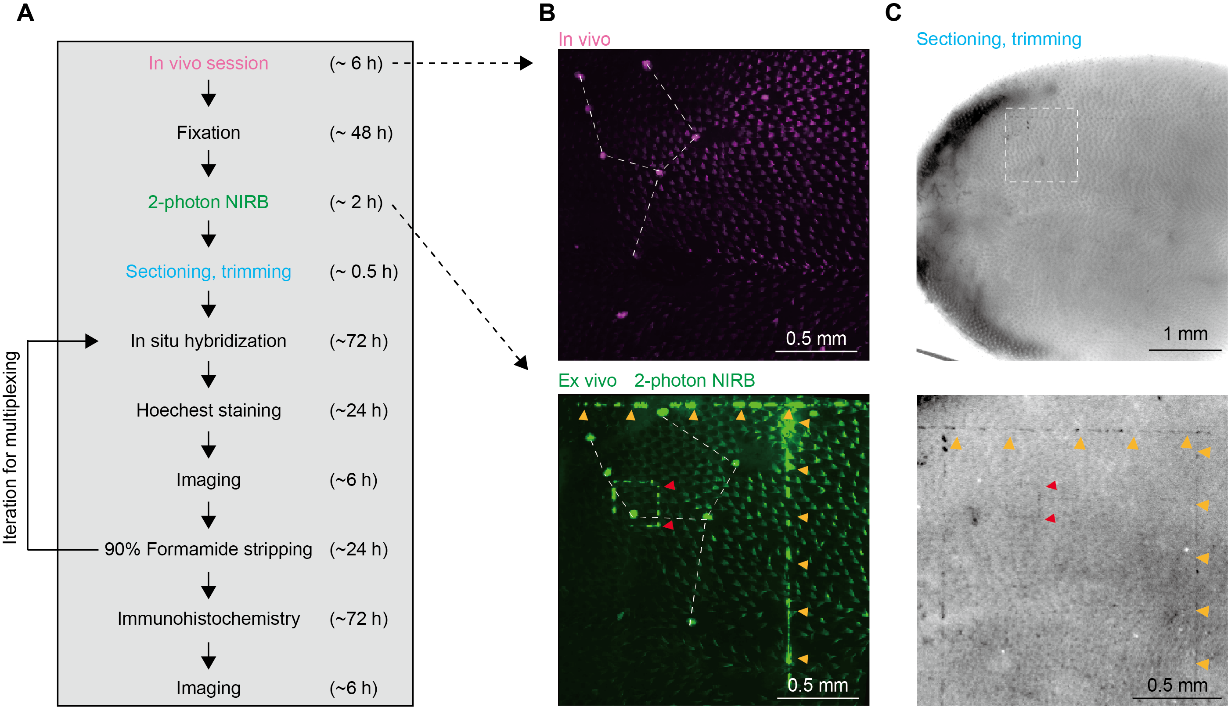


**Figure S2 Scheme of the correlative platform**

**(A)** Schematic illustration of the workflow for the correlative platform. **(B)** Representative of an in vivo large field-of-view-image (top) and a corresponding ex vivo image after NIRB marking (bottom). The white dashed line indicate identical taste buds observed in both in vivo and ex vivo. Scale bar: 0.5 mm **(C)** Surgical microscopic view after NIRB marking (top) along with a magnified image of the region of interest. Yellow arrowheads indicate macroscale NIRB marks for trimming, while red arrowheads denote microscale NIRB marks identifying individual taste buds, corresponding to the arrow indicators in the lower image of (B). Scale bar: 1 mm (top), 0.5 mm (bottom).


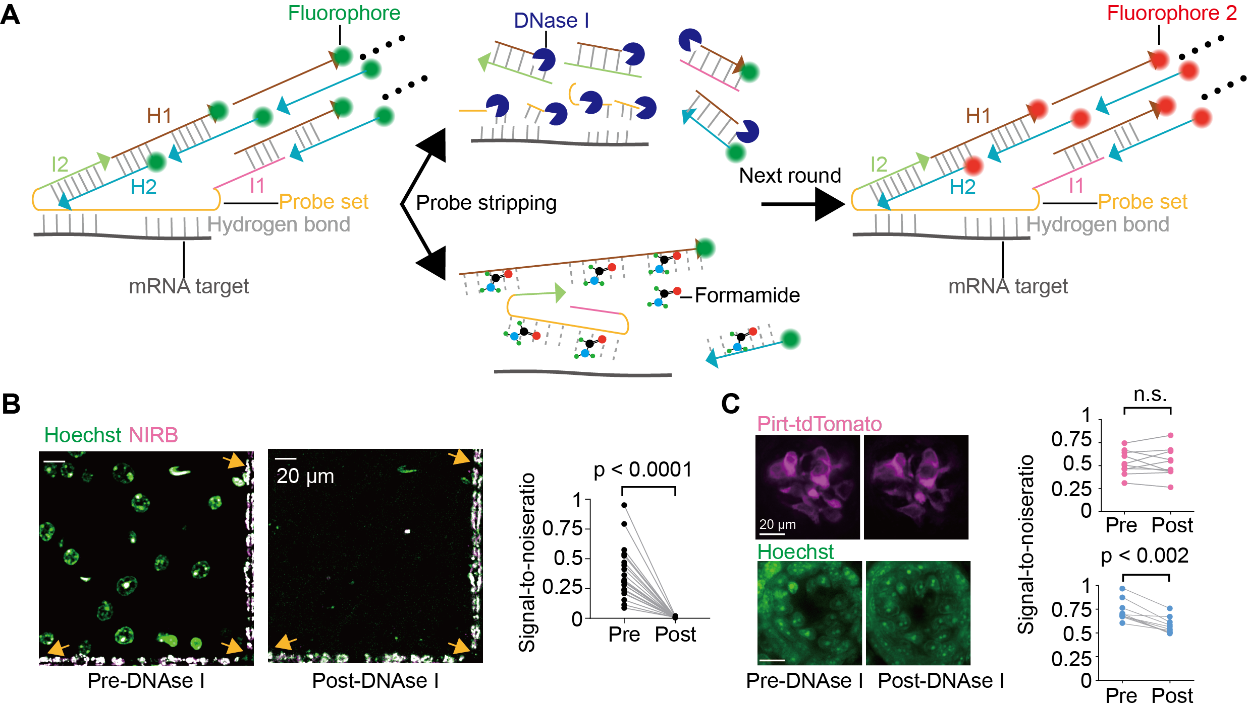


**Figure S3. Comparison of DNase I effects on brain and tongue**

**(A)** Schematic illustration of DNase I and formamide stripping mechanism. **(B)** Representative images showing brain tissue before and after conventional DNase I treatment. The Hoechst signal significantly decreases following DNase I treatment. Yellow arrow denotes NIRB. Scale bar: 20 µm. **(C)** Representative images of tongue tissue before and after conventional DNase I treatment. While the fluorescence reporter (tdTomato) intensity remained statistically unchanged (left), the Hoechst signal showed a statistically significant decrease. However, the structural features are well preserved compared to brain tissue (right). Scale bar: 20 µm. The Mann-Whitney paired U-test was used for statistical analysis of intensity changes.


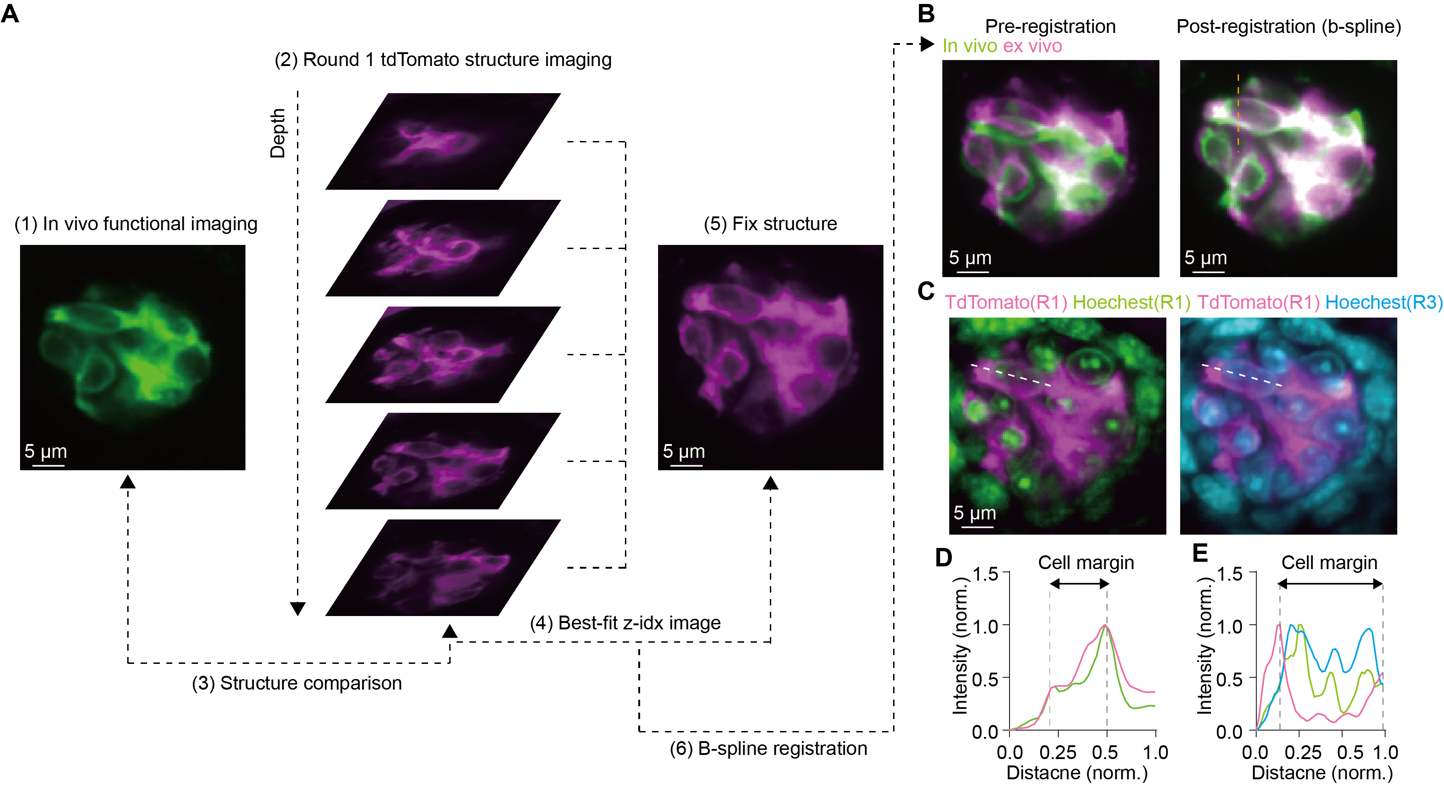


**Figure. S4 Schematic representation of steps for image registration**

**(A)** Schematic illustration for in vivo – ex vivo image registration. Moving image is acquired by 100 – 1,000 frames average during in vivo functional imaging. The fixed image is selected among the tdTomato volumetric images from round 1, which have the highest image correlation index. In some cases, 2–3 z-stacks from the round 1 tdTomato images are projected for the fixed image. **(B)** Comparison of images between before and after B-spline registration. **(C)** After performing rigid registration of the round 3 Hoechst signal to the round 1, the resulting round 3 Hoechst image is merged with the round 1 tdTomato image. **(D-E)** Line profiles of fluorescence intensities along the yellow-dashed line in (B) after non-rigid registration and along the white-dashed line in (C) after rigid registration. The colored lines denote each imaging round: (D) green: in vivo tdTomato, magenta: round 1 tdTomato; (E) magenta: round 1 tdTomato, green: round 1 Hoechst, cyan: round 3 Hoechst after rigid registration.


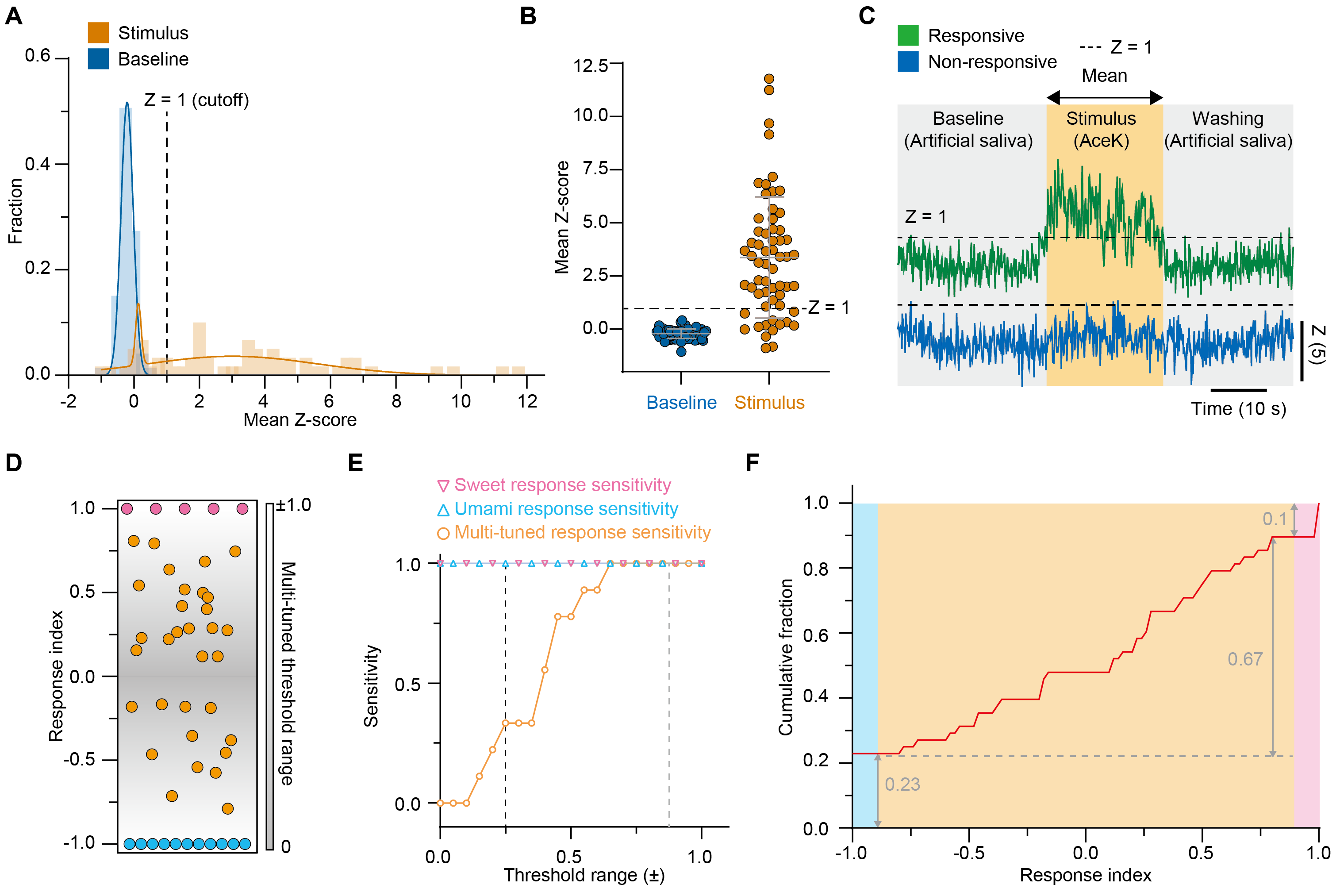


**Figure S5. Determination of optimal thresholds for response detection and multi-tuning classification**

**(A)** Mean Z-score histogram during artificial saliva (baseline, blue) or tastant stimulation (green). A cutoff of Z = 1 was applied, corresponding to the threshold that discriminates baseline calcium activity. **(B)** Scatter plot of the data shown in (A). Note that the cutoff point (Z = 1, black dashed line) allowed reliable discrimination of baseline activity, and was therefore used to determine the presence or absence of responses. **(C)** Representative Ca²⁺ time traces of afferent nerves. The baseline period refers to the initial 20 s the artificial saliva was applied. Nerves were classified as responsive (top, green trace) when the Z-score during stimulation exceeded 1 (black dashed line), or as non-responsive (bottom, blue trace) when the Z-score remained below 1. Shaded light gray indicates the period of artificial saliva delivery, and yellow denotes the period of tastant stimulation **(D)** Plot of response indexes in individual nerves. Colored dots denote the classified response types. Gray shading indicates the range of response index thresholds used for multi-tuned classification. **(E)** Sensitivity of classification across response index thresholds. Note that single-tuned responses showed near-perfect sensitivity (=1) regardless of threshold, while multi-tuned sensitivity was strongly threshold-dependent. **(F)** Cumulative distribution function (CDF) curve of the response index in (D).


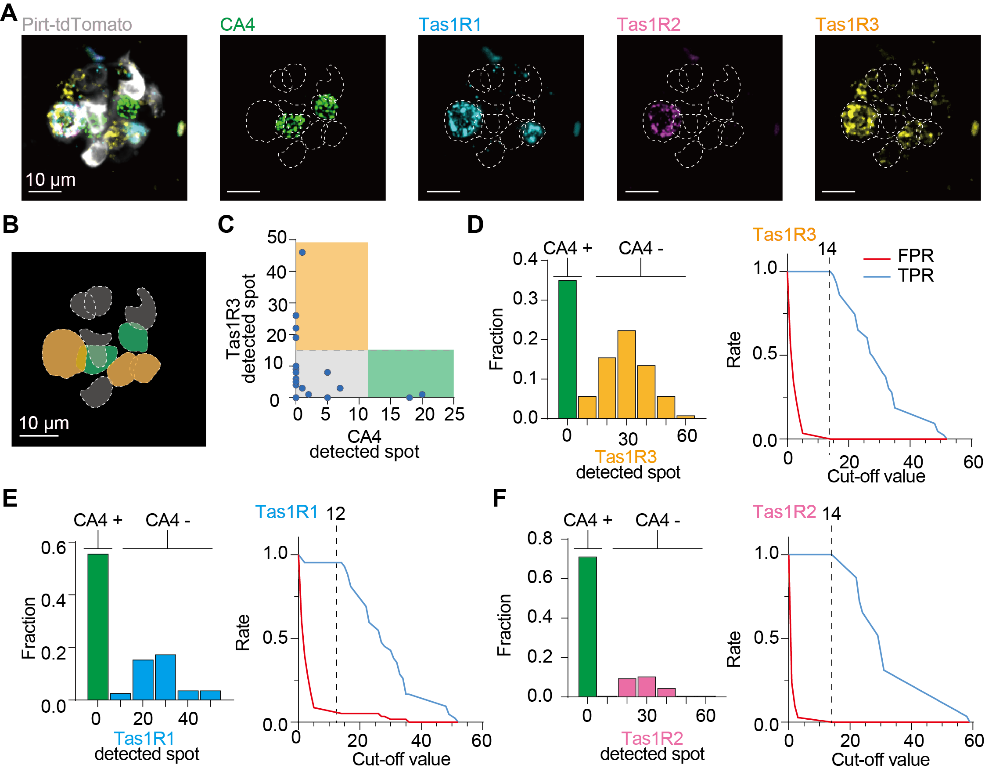


**Figure S6. Method for determining mRNA positivity/negativity using CA4+ Cells**

**(A)** Representative images showing multiplexed smFISH results. The detected Tas1Rs smFISH spots are nearly zero in cells determined as CA4+ cells. Each color represents a distinctly targeted smFISH probe (green: CA4, cyan: Tas1R1, magenta: Tas1R2, yellow: Tas1R3). **(B)** A false-color image showing Tas1R3+ cells (yellow), CA4+ cells (green), and cells in which nothing is detected with these probe set (gray) in (A). White-dashed circles indicate individual cell margins. **(C)** Spot counts of cells with expression of Tas1R3 and CA4. The colors of each quadrant correspond to the colors in (B). **(D-F)** Histograms showing Tas1R3, Tas1R1, and Tas1R2 spot detection in individual cells. CA4+ (Tas1R-negative) cells are represented in green bars and CA4- (Tas1R-positive) in yellow (Tas1R3), cyan (Tas1R1), and magenta (Tas1R2). Identification of optimal cut-off values based on cells with detected spots which are Tas1R-positive and CA4-negative. Optimal thresholds for classification were determined at 14 spots for Tas1R3 and Tas1R2, and 12 spots for Tas1R1.


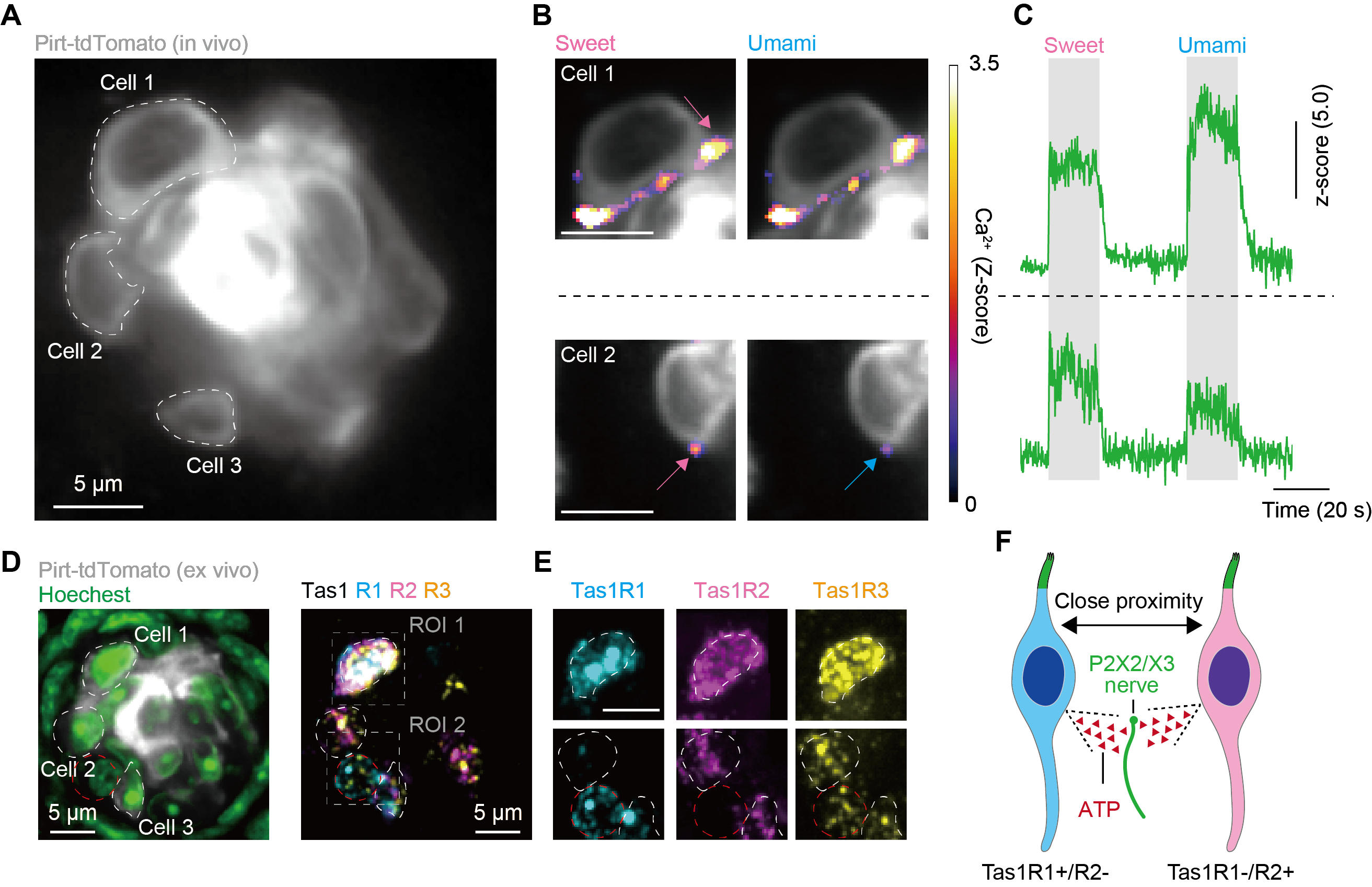


**Figure S7. Close proximity mediated dual-tuned response**

**(A)** Representative image of Pirt-tdTomato taste bud. **(B)** Images of taste cells showing dual-tuned responses of afferent nerves to sweet and umami taste stimuli in (A). **(C)** Nerve calcium traces corresponding to images in (B). **(D)** Images of the taste bud in (A) visualized utilizing the correlative platform, showing tdTomato (gray) and Hoechst (green) staining (left). Tas1Rs mRNAs are visualized using smFISH (right). The merged image shows the smFISH signals for each receptor (cyan: Tas1R1, magenta: Tas1R2, yellow: Tas1R3). The cell labeled with the red-dashed margin refers to a cell that was tdTomato-negative, compared to ones with the white-dashed margin, which were tdTomato-positive. **(E)** Magnified view of the image in (D). Taste cells in close proximity to the dual-tuned afferent nerves shown in (B). **(F)** Schematic representation of dual-tuned afferent nerve responses caused by ATP spillover from taste cells in close proximity (ROI 2).


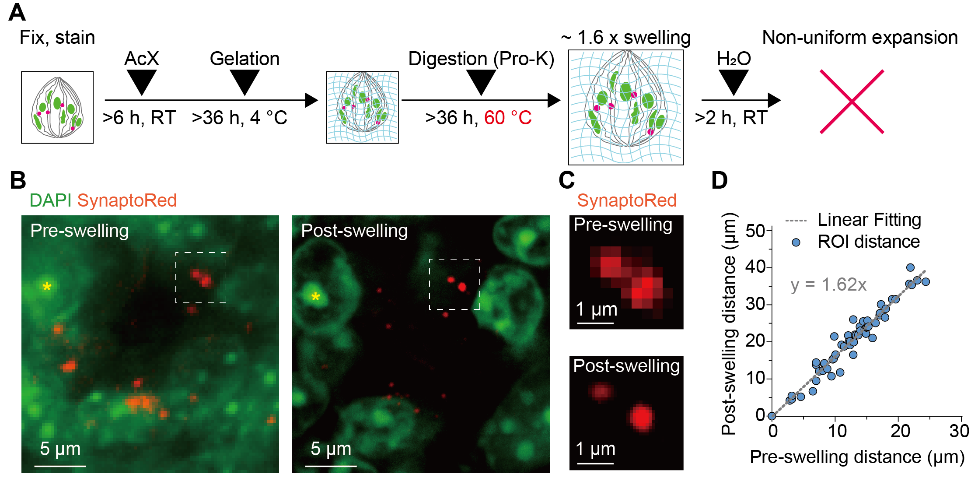


**Figure. S8. Expansion process for tongue tissue**

**(A)** Illustration describing the workflow of the tongue expansion technique. The final 4X expansion step was not performed due to excessive distortion. **(B)** Representative images comparing pre- and post-tissue swelling. SynaptoRed was administered via intraperitoneal injection one day prior to mouse sacrifice, labeling synaptic vesicles. Hoechst was stained the day before AcX treatment. Yellow asterisks indicate the same heterochromatin structure. Scale bar in the post-swelling image represents an actual size of 8 µm. **(C)** Magnified images of the region marked with a white-dashed square in (B), showing two blurred spots that are spatially resolved. **(D)** Measurement of the 3D distance at the same location pre- and post-swelling.

Table S1. Comparison of existing experimental and molecular assay

|  | **Methods for taste research** | | | | |
| --- | --- | --- | --- | --- | --- |
|  | scRNA seq | In vitro | Ex vivo | In vivo (μTongue) | Correlative platform |
| Functional information | None | Low | Moderate | High | High |
| Molecular information | High | High | High | Limited | Moderate |
| Spatial information | None | None | Moderate | High | High |
| Reference | ^[1]^ | ^[2]^ | ^[3]^ | ^[4]^ | N/A |

Table S2. Reagents for artificial saliva and tastant

| **Reagent** | **Components** |
| --- | --- |
| Artificial saliva | Sodium chloride (S9888, Sigma-Aldrich) |
|  | Potassium chloride (P3911, Sigma-Aldrich) |
|  | Sodium bicarbonate (S6014, Sigma-Aldrich) |
|  | Potassium bicarbonate (237205, Sigma-Aldrich |
|  | Calcium chloride (21115, Sigma-Aldrich) |
|  | Magnesium chloride (M8266, Sigma-Aldrich) |
|  | Potassium phosphate dibasic (P3786, Sigma-Aldrich) |
|  | Potassium phosphate monobasic (P0662, Sigma-Aldrich) |
|  | Hydrochloric acid (320331, Sigma-Aldrich) |
| Sweet taste | Acesulfame K (AceK) (04054, Sigma-Aldrich) |
| Umami taste | IMP (57510, Sigma-Aldrich) |
| Umami taste | MPG (49601, Sigma-Aldrich) |
| Sour taste | Citric acid (C0759, Sigma-Aldrich) |
| Sour taste | Acetic acid (A6283, Sigma-Aldrich) |

**Table S3. HCR probes and oligonucleotides**

Note that the total rounds are marker-genes used for taste cell experiments. All probes listed below are next-generation hairpin chain reaction (HCR v2.0) from Molecular Instruments.

| **Genes** | **Accession #** | **HCR hairpin** | **Fluorophores** |
| --- | --- | --- | --- |
| CA4 | NM_007607.3 | B1, 4 | AF647 |
| Tas1R1 | NM_031867.2 | B2 | AF647 |
| Tas1R2 | NM_031873.1 | B1 | AF488 |
| Tas1R3 | NM_031872.2 | B4 | AF647 |
| **Amplifier** | | | |
| HCR amplifier B1 | Molecular Instrument (N/A) | | |
| HCR amplifier B2 | Molecular Instrument (N/A) | | |
| HCR amplifier B4 | Molecular Instrument (N/A) | | |

**Table S4. Reagent for Immunofluorescence**

| **Reagent** | **Components** |
| --- | --- |
| Primary antibody | RFP (600-401-379, ROCKLAND) |
|  | Carbonic Anhydrase IV/CA4 (AF2414, R&D system) |
|  | P2X3 (APR-016, Alomone labs) |
|  | guinea pig anti-mTRPM5 (Taste Research Center, Yonsei University) |
| Secondary antibody | Goat-anti-Rabbit CF633 (20123, Biotium) |
|  | Donkey-anti-Goat-AF488 (A-11055, ThermoFisher) |
|  | ­Donkey-anti-Goat-AF647 (A-21477, ThermoFisher) |
|  | Goat-anti-Guinea pig-AF488 (A-11073, ThermoFisher) |
|  | Goat-anti-Guinea pig-AF647 (A-21450, ThermoFisher) |
|  | Goat-anti-Rabbit-AF488 (A-11008, ThermoFisher) |
| Blocking buffer | CAS-Block (008120, ThermoFisher) |
| Washing buffer | 0.1% Triton X-100 in 1X PBS |

**Table S5. Software used in this study**

| **Purpose** | **Software** | **Source** |
| --- | --- | --- |
| Image visualization | Fiji – ImageJ 2.3.0 | NIH |
| Image stitching | BigStitcher | https://imagej.net/plugins/bigstitcher |
|  | NIS-Stiching | NIS-Elements (Nikon) |
| Motion correction | NoRMCorre | ^[5]^ |
| B-spline registration | ITK-Elastix | ^[6]^ |
| Rigid body registration | MATLAB 2021b | MathWorks |
| Cell segmentation | VAST_Lite_1.4.0 | ^[7]^ |
| dHCR spot count | Airlocalize | ^[8]^ |
| Statics and graph generation | GraphPad Prism 9 | GraphPad |

**REFERENCE**

[1] S. K. Sukumaran, B. C. Lewandowski, Y. Qin, R. Kotha, A. A. Bachmanov, R. F. Margolskee, *Sci. Rep.* **2017**, *7*, 1.

[2] A. Taruno, V. Vingtdeux, M. Ohmoto, Z. Ma, G. Dvoryanchikov, A. Li, L. Adrien, H. Zhao, S. Leung, M. Abernethy, J. Koppel, P. Davies, M. M. Civan, N. Chaudhari, I. Matsumoto, G. Hellekant, M. G. Tordoff, P. Marambaud, J. K. Foskett, *Nature* **2013**, *495*, 223.

[3] J. K. Roebber, S. D. Roper, N. Chaudhari, *J. Neurosci.* **2019**, *39*, 6224.

[4] G. Y. Park, G. Lee, J. Yoon, J. Han, P. Choi, M. Kim, S. Lee, C. Park, Z. Wu, Y. Li, M. Choi, *Cell* **2024**, 1.

[5] E. A. Pnevmatikakis, A. Giovannucci, *J. Neurosci. Methods* **2017**, *291*, 83.

[6] K. Ntatsis, N. Dekker, V. van der Valk, T. Birdsong, D. Zukić, S. Klein, M. Staring, M. McCormick, *Proc. 22nd Python Sci. Conf.* **2023**, 101.

[7] D. R. Berger, H. S. Seung, J. W. Lichtman, *Front. Neural Circuits* **2018**, *12*.

[8] T. Lionnet, K. Czaplinski, X. Darzacq, Y. Shav-Tal, A. L. Wells, J. A. Chao, H. Y. Park, V. De Turris, M. Lopez-Jones, R. H. Singer, *Nat. Methods* **2011**, *8*, 165.
